# LI-RADS v2018 versus KLCA-NCC v2022: comparison of probability-based HCC categories

**DOI:** 10.1007/s00330-026-12696-0

**Published:** 2026-06-26

**Authors:** Jeong Hee Yoon, Eun Sun Lee, Young Kon Kim, Chang Hee Lee, Jeong Woo Kim, Won Chang, Joon-Il Choi, Beom Jin Park, Jin-Young Choi, Seung-seob Kim, Jeong-Sik Yu, Seong Jin Park, Myung-Won You, Myoung-jin Jang, Hee Sun Park, Jeong Min Lee

**Affiliations:** 1 Department of Radiology, Seoul National University Hospital, Seoul, 03080 Republic of Korea; 2 Department of Radiology, Seoul National University College of Medicine, Seoul, 03080 Republic of Korea; 3 Department of Radiology, Chung-Ang University Hospital, Seoul, 06973 Republic of Korea; 4 Department of Radiology and Center for Imaging Science, Samsung Medical Center, Sungkyunkwan University School of Medicine, Seoul, 06351 Republic of Korea; 5 Department of Radiology, Korea University Guro Hospital & College of Medicine, Seoul, 08380 Republic of Korea; 6 Department of Radiology, Seoul National University Bundang Hospital, Seongnam, 13620 Republic of Korea; 7 Department of Radiology, Seoul Saint Mary’s Hospital, College of Medicine, The Catholic University of Korea, 06591 Seoul, Republic of Korea; 8 Department of Radiology and Advanced Medical Imaging Institute, Korea University Anam Hospital, Korea University College of Medicine, Seoul, 02841 Republic of Korea; 9 Department of Radiology and Research Institute of Radiological Science, Severance Hospital, Yonsei University College of Medicine, Seoul, 03722 Republic of Korea; 10 Department of Radiology, Gangnam Severance Hospital, Yonsei University College of Medicine, Seoul, 06273 Republic of Korea; 11 Department of Radiology, CHA University Bundang Medical Center, Seongnam, 13496 Republic of Korea; 12 Department of Radiology, Kyung Hee University College of Medicine, Kyung Hee University Hospital, Seoul, 02447 Republic of Korea; 13 Medical Research Collaborating Center, Seoul National University Hospital & Seoul National University College of Medicine, Seoul, 03080 Republic of Korea; 14 Department of Radiology, Konkuk University Medical Center, Konkuk University School of Medicine, 120-1 Neungdong-ro, Gwangjin-gu, Seoul, 05030 Republic of Korea; 15 Institute of Radiation Medicine, Seoul National University Medical Research Center, Seoul, 03080 Republic of Korea

**Keywords:** Carcinoma, Hepatocellular, Gadolinium DTPA, Predictive value of tests, Sensitivity and specificity

## Abstract

**Objectives:**

To compare the diagnostic performance of corresponding probability-based categories between LI-RADS v2018 and Korean Liver Cancer Association-National Cancer Center (KLCA-NCC) v2022.

**Materials and methods:**

This retrospective multicenter study included treatment-naïve patients at risk of hepatocellular carcinoma (HCC) who underwent gadoxetic acid-enhanced MRI between January 2015 and June 2018. Four radiologists independently evaluated MRI and CT when available; observations were categorized according to both criteria. Reference standards were pathology or clinical diagnosis. Generalized estimating equations were used for lesion clustering.

**Results:**

The study included 2237 patients (1666 men; mean age, 59 ± 11 years) with 2445 lesions. On MRI, LR-5 showed a higher positive predictive value (PPV) than KLCA-NCC “definite HCC” (93.9% vs. 91.7%, *p* < 0.001). PPV was not different between LR-4 and KLCA-NCC “probable HCC” (68.2% vs. 64.1%, *p* = 0.141). HCC proportion was 13.2% in LR-3 and 40.8% in KLCA-NCC “indeterminate nodule” (*p* < 0.001). In 1455 patients with 1590 lesions on CT, LR-5 and KLCA-NCC had comparable PPV (92.9% in each). PPV of KLCA-NCC “probable HCC” was higher than LR-4 (83.8% vs. 73.9%, *p* = 0.012). HCC proportion was 52.0% in LR-3 and 46.2% in KLCA-NCC “indeterminate nodule” (*p* = 0.001).

**Conclusion:**

Both LI-RADS v2018 and KLCA-NCC v2022 demonstrated effective probability-based risk stratification, but corresponding probability categories were not interchangeable. On CT, category performance differed from MRI, highlighting the need for modality-specific refinement to improve diagnostic precision.

**Key Points:**

***Question*** *Do conceptually corresponding diagnostic categories in LI-RADS v2018 and KLCA-NCC v2022 demonstrate equivalent diagnostic performance for HCC?*

***Findings*** *Both systems demonstrated effective probability-based stratification, but corresponding categories were not equivalent, and the performance was modality-specific*.

***Clinical relevance*** *Corresponding categories are not interchangeable between the two systems. The heterogeneity within “probable HCC” and modality-specific performance differences suggest potential benefits from subcategorization and refinement of CT-specific criteria.*

**Graphical Abstract:**

**Figure:**
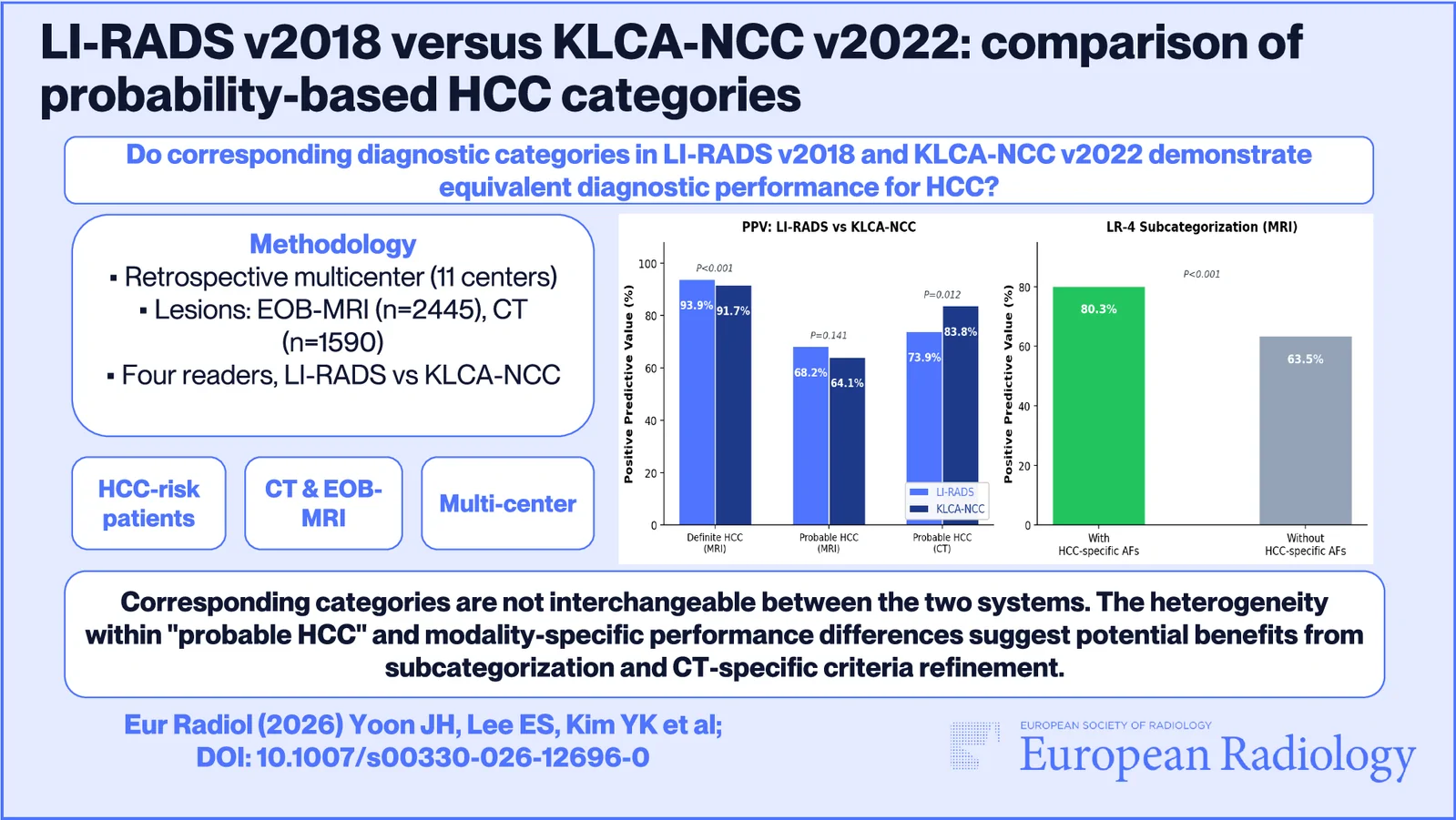

## Introduction

Hepatocellular carcinoma (HCC) is the most common primary liver cancer, accounting for 75–85% of cases [1]. The diagnosis of HCC typically depends on distinctive imaging characteristics, namely non-rim arterial-phase hyperenhancement (APHE) and non-peripheral washout. Traditionally, most guidelines have used binary diagnostic algorithms such as either HCC or not HCC. However, recent advancements have introduced more nuanced categorization systems that assess the likelihood of HCC [2–6].

The Liver Imaging Reporting and Data System (LI-RADS) v2018 and the Korean Liver Cancer Association-National Cancer Center (KLCA-NCC) v2022 guidelines now categorize hepatic observations based on the probability of HCC [5, 7]. In LI-RADS v2018, four major features, including non-rim APHE, non-peripheral portal or delayed washout, enhancing capsule (EC), and threshold growth, are used to categorize observations from LR-3 to LR-5 in combination with size [7]. Ancillary features (AFs) suggesting malignancy or benign may optionally adjust the category by one level. Similarly, KLCA-NCC v2022 uses two major features of non-rim APHE and non-peripheral washout, namely the transitional phase (TP) and hepatobiliary phase (HBP), for gadoxetic acid-enhanced MRI [5]. In observations not meeting two major features, “probable HCC” requires non-rim APHE plus at least one AF suggesting malignancy/HCC-specific, or the absence of APHE with both malignancy-suggestive and HCC-specific AFs. Key differences include a) extended washout timing in KLCA-NCC to include TP and HBP; b) EC and threshold growth are AFs in KLCA-NCC; and c) incorporation of benignity-favoring AFs in LI-RADS but not in KLCA-NCC.

Both systems include categories for “definite HCC” (LR-5 in LI-RADS and “definite HCC” in KLCA-NCC), “probable HCC” (LR-4 in LI-RADS and “probable HCC” in KLCA-NCC) and “indeterminate for malignancy nodule” (LR-3 in LI-RADS) and “indeterminate nodule” in KLCA-NCC. Although they correspond to conceptually similar probability categories in each system, the evidence supporting their equivalence is limited due to distinct diagnostic criteria. Despite the critical role of accurate categorization in management, there is a notable lack of studies comparing those categories between the two systems. Therefore, this study aims to compare the probability of HCC across corresponding LI-RADS v2018 and KLCA-NCC v2022 categories.

## Materials and methods

Eleven tertiary hospitals in South Korea participated in this retrospective multicenter study. The institutional review boards of each institution waived the requirement for informed consent. This work received financial support from Bayer; however, the authors maintained complete control over the data and information submitted for publication at all times.

Data have been published elsewhere comparing four HCC diagnosis guidelines [8], measuring the gain of second-line examination [9], and comparing KLCA-NCC v2018 and v2022 [10]. The same eligibility criteria were applied in this study. Briefly, these criteria included: a) HCC-naïve patients with chronic hepatitis B or C, or cirrhosis of any etiology; b) patients who underwent gadoxetic acid-enhanced MRI between January 2015 and June 2018; c) those with at least one non-cystic liver lesion ( ≥ 10 mm); d) lesions that were pathologically diagnosed; or e) benign lesions with characteristic imaging features on contrast-enhanced ultrasound, CT, and/or MRI, and stable lesion size for more than 2 years or a decrease on follow-up imaging. Exclusion criteria were as follows: a) absence of essential MRI sequences; b) multiple ( ≥ 6) lesions; c) benign lesions lacking confirmatory evidence; d) malignant lesions not pathologically confirmed within 3 months of MRI; and e) lesions with inconclusive pathology. Among the eligible patients with MRI, we identified those with available CT scans for subgroup analyses, using the following criteria: a) liver CT performed within 8 weeks of the MRI with malignant lesions; b) not receiving any treatment between CT and MRI; and c) demonstrating no rapid disease progression or regression between scans.

### Image acquisition

MRI scans were performed using either 1.5 T or 3 T. The examinations incorporated heavily T2-weighted images, T2-weighted images, diffusion-weighted images with at least two *b*-values ranging from 0 to 1000 s/mm^2^, in- and opposed-phases, and precontrast, arterial, portal, transitional, and hepatobiliary phases. A standard dose of gadoxetic acid (0.025 mmol/kg, Eovist or Primovist, Bayer) was administered. Detailed scan parameters are in the Supplement.

CT scans were obtained using multi-detector scanners equipped with 4–256 channels, operating at 80–140 kVp in accordance with the protocols of each institution. The examination consisted of precontrast, arterial, portal venous, and delayed phases. Iodine contrast medium, dosed at 1.5–1.6 mL/kg, was administered (Supplement).

### Image analysis

Details of the image analysis have been described elsewhere [8]. Four fellowship-trained body radiologists (J.H.Y., J.W.K., S.K., M.Y., with 11, 5, 7, and 9 years of experience, respectively) independently reviewed the index tumors in all patients on a web-based platform, while blinded to the clinical information and diagnoses except for patients belonging to the HCC high-risk group [8]. Major features, LR-M features, the presence of tumor-in-vein, and AFs were assessed, except threshold/subthreshold growths, ultrasound visibility as a discrete nodule, and size stability for ≥ 2 years or reduction [8]. The LI-RADS category was automatically assigned, while KLCA-NCC v2022 categories (“definite HCC,” “probable HCC,” “indeterminate nodule,” and “not applying criteria”) were re-assigned based on individual patients’ data because the web-based platform was built before releasing KLCA-NCC v2022 [5] (Supplement).

### Reference standard

All malignant lesions were histologically confirmed within 90 days following gadoxetic acid-enhanced MRI. Benign lesions were diagnosed either by histology or by demonstrating characteristic imaging features (Supplement), along with stable or decreased size on follow-up imaging over a period of 2 years.

### Statistical analysis

The pooled analysis across four readers was the pre-specified primary analysis, and per-reader analyses are provided together. Given the correlations among multiple observations in the same patients, the diagnostic performance of two guidelines was assessed using generalized estimating equations (GEE) with a binomial distribution and logit link function [11]. The GEE analysis used an independent working correlation and a robust sandwich variance estimator to produce unbiased standard error estimates even when the assumed correlation structure was incorrectly specified. Interobserver agreement for the classifications “definite HCC” and “probable HCC” according to both guidelines was evaluated using Fleiss kappa and Gwet’s agreement coefficient (AC1) [12, 13].

The diagnostic performance of each guideline was evaluated based on sensitivity, specificity, positive predictive value (PPV), and negative predictive value (NPV). All statistical analyses were conducted using SPSS (ver. 27, IBM) and SAS 9.4 (SAS Institute Inc.). A *p*-value of less than 0.05 was considered statistically significant.

## Results

**Fig. 1:**
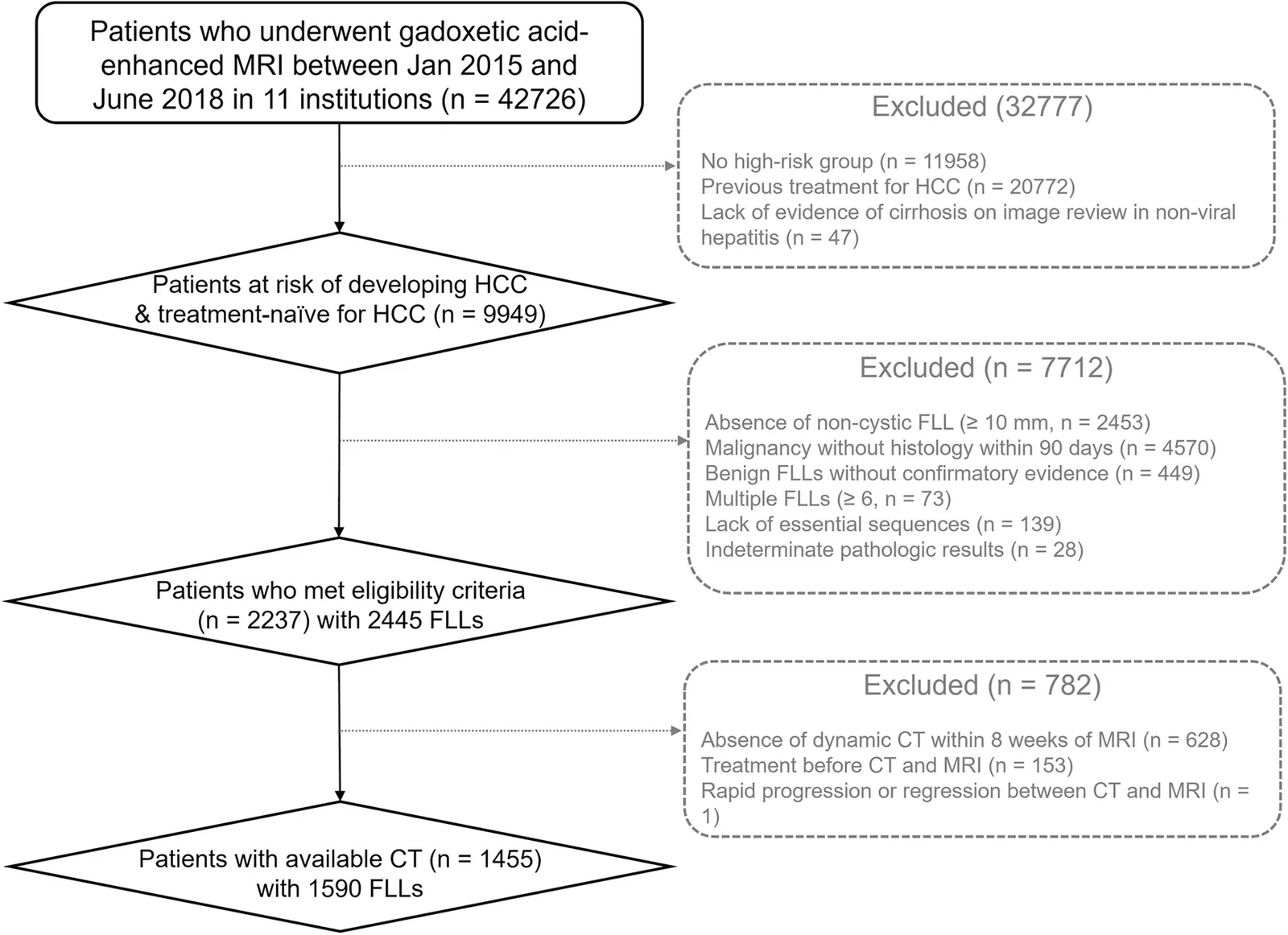
Study flow. FLL, focal liver lesion; HCC, hepatocellular carcinoma

Among the 42726 patients who underwent gadoxetic acid-enhanced MRI from January 2015 to June 2018, 2237 eligible patients (1666 men, mean age 59 ± 11 years) with 2445 lesions (median size 27.4 mm, IQR 17.8–46.8 mm) were included in the analysis (Fig. 1). Patients had one (*n* = 2044), two (*n* = 178) and three index tumors (*n* = 15). Further details are described in Table 1 and Supplement. Of these, 1455 patients (1101 men, mean age 59 ± 12 years) with 1590 lesions (median size, 22.6 mm, IQR 17.5–44.8 mm) who had undergone CT were included in a subgroup analysis (Table S1). The median interval between CT and MRI was 11 days (IQR: 5–21 days).

**Table 1:** Demographic characteristics of the study participants

Variables	Values
Sex (male: female)	1666:571
Age (years)	59 ± 11 (19–97)
Men	59 ± 11 (19–89)
Women	60 ± 12 (20–97)
Underlying liver disease	
Chronic hepatitis B	1755 (78.5)
Non-viral cirrhosis^a^	334 (14.9)
Chronic hepatitis C	112 (5.0)
Co-infection of chronic hepatitis B and C viruses	22 (1.0)
Chronic hepatitis B and alcohol	14 (0.6)
Child-Pugh classification	
Chronic liver disease	972 (43.5)
Class A	1122 (50.2)
Class B	121 (5.4)
Class C	22 (1.0)
Diagnostic confirmation	
Hepatic resection	1598 (71.4)
Biopsy	467 (20.9)
Clinical follow-up	172 (7.7)
Interval between MRI and histologic confirmation (days)	
Benign and malignant lesions (*n* = 2060)	16.7 ± 15.4 (0–90)
Benign lesions (*n* = 5)^b^	368.6 ± 260.5 (97–748)
Diagnosis and size of liver lesions (*n* = 2445 lesions)	
HCC (*n* = 1694)	28.6 mm (10–202.1)
Non-HCC malignancy (*n* = 364)	42.8 mm (10.2–189.4)
Benign (*n* = 387)	16.6 mm (10–110.3)

Values are mean ± standard deviation (range), median (range) or number (percentage)

^a^ Cryptogenic (*n* = 184), alcoholic (*n* = 82), non-alcoholic steatohepatitis (*n* = 45), biliary (*n* = 18), autoimmune hepatitis (*n* = 3), Wilson disease (*n* = 2)

^b^ Meeting the criteria of benign lesions (typical features and presence of clinical follow-up) and histologically confirmed 90 days or more after MRI

### Categorization of hepatic observations according to guidelines

On MRI, lesions were categorized into LR-NC (*n* = 19), LR-2 (*n* = 766), LR-3 (*n* = 296), LR-4 (*n* = 1295), LR-5 (*n* = 5091) and LR-M (*n* = 2313) according to the pooled data of four readers. With KLCA-NCC, lesions were classified as “definite HCC” (*n* = 5776), “probable HCC” (*n* = 582), “indeterminate nodule” (*n* = 598) and “not applying diagnostic criteria” (*n* = 2824). On CT, observations were LR-2 (*n* = 388), LR-3 (*n* = 790), LR-4 (*n* = 433), LR-5 (*n* = 3729) and LR-M (*n* = 1020) while KLCA-NCC v2022 classified them as “definite HCC” (*n* = 3677), “probable HCC” (*n* = 99), “indeterminate nodule” (*n* = 1681) and “not applying diagnostic criteria” (*n* = 903).

### Comparison of the diagnostic performance of “definite and probable HCC” between guidelines

When both “definite HCC” and “probable HCC” were regarded as positive test results, no difference was observed between LI-RADS and KLCA-NCC in sensitivity (83.6% vs. 83.7%, *p* = 0.779) and specificity (75.9% vs. 77.1%, *p* = 0.215) on MRI (Tables S2 and S3). The PPV was 88.7% in LI-RADS and 89.2% in KLCA-NCC (*p* = 0.206). On CT, LI-RADS showed higher sensitivity (81.6% vs. 75.5%) and lower specificity (78.0% vs. 84.0%) than KLCA-NCC (*p* < 0.001 for both).

### Comparison of the performance of each category between guidelines

**Fig. 2:**
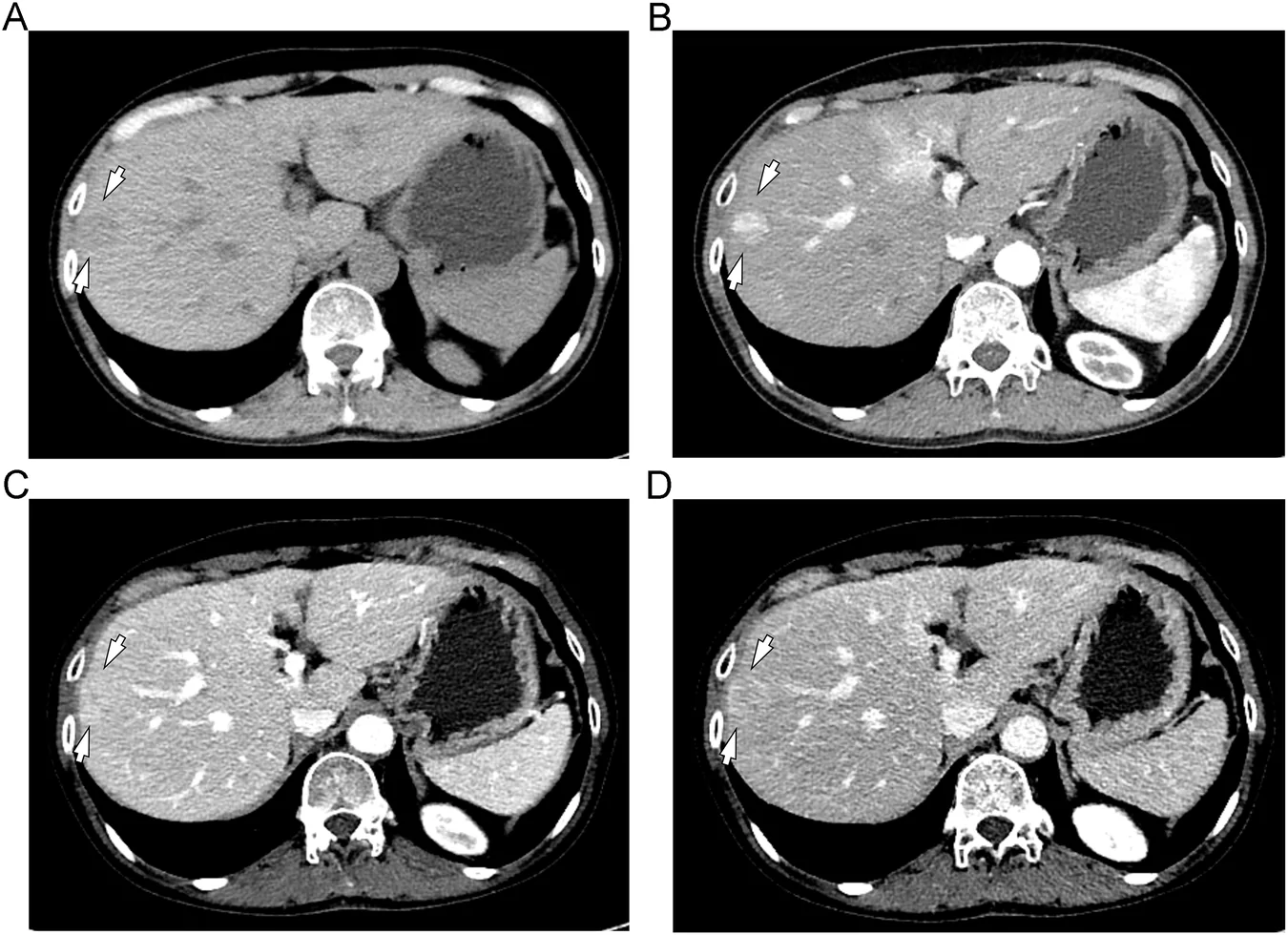
A 63-year-old female patient with chronic hepatitis B and surgically confirmed HCC. **A** On CT, a 21.3 mm mass (arrows) in segment 8 has **B** non-rim APHE, **C** no portal washout, and **D** enhancing capsule (EC) without delayed washout. The mass is classified as LR-5 according to LI-RADS v2018 by fulfilling two major features (**B**, **D**). In KLCA-NCC v2022, this mass is “probable HCC” because it lacks non-peripheral washout, which is required for “definite HCC”; EC serves as an AF for “probable HCC”. HCC, hepatocellular carcinoma; APHE, arterial phase hyperenhancement; LI-RADS, Liver Imaging Reporting and Data System; KLCA-NCC, Korean Liver Cancer Association-National Cancer Center; AF, ancillary feature

*“definite HCC”—*On MRI, KLCA-NCC “definite HCC” showed higher sensitivity than LR-5 (78.2% vs. 70.5%, *p* < 0.001) while specificity was higher in LR-5 (89.6% vs. 84.1%, *p* < 0.001) (Tables 2 and S4). On CT, LR-5 showed higher sensitivity than KLCA-NCC “definite HCC” (74.7% vs. 73.7%, *p* < 0.001), with comparable PPV (92.9% in each, *p* = 0.404) (Fig. 2).

**Table 2:** Comparison between the diagnostic performance of LR-5 in LI-RADS v2018 and “definite HCC” in KLCA-NCC v2022 on MRI

Guidelines	Sensitivity (%)	Specificity (%)	PPV (%)	NPV (%)
MRI (*n* = 2445)				
LI-RADS v2018	70.5 (4780/6776) [68.8, 72.3]	89.6 (2693/3004) [87.8, 91.2]	93.9 (4780/5091) [92.7, 94.9]	57.4 (2693/4689) [54.7, 60.1]
KLCA-NCC v2022	78.2 (5297/6776) [76.6, 79.7]	84.1 (2525/3004) [81.9, 86.0]	91.7 (5297/5776) [90.4, 92.8]	63.1 (2525/4004) [63.0, 65.8]
Difference	−7.6 [−8.7, −6.5]	5.6 [4.3, 6.9]	2.2 [1.5, 2.9]	−5.6 [−6.8, −4.5]
* p*-value	< 0.001	< 0.001	< 0.001	< 0.001
CT (*n* = 1590)				
LI-RADS v2018	74.7 (3463/4636) [72.6, 76.7]	84.6 (1458/1724) [81.6, 87.1]	92.9 (3463/3729) [91.3, 94.1]	55.4 (1458/2631) [51.8, 58.9]
KLCA-NCC v2022	73.7 (3416/4636) [71.6, 75.7]	84.9 (1464/1724) [82.0, 87.4]	92.9 (3416/3676) [91.4, 94.2]	54.5 (1464/2684) [51.0, 58.1]
Difference	1.0 [0.7, 1.3]	−0.3 [−0.7, 0.0]	−0.1 [−0.2, 0.1]	0.9 [0.5, 1.2]
*p*-value	< 0.001	0.033	0.404	< 0.001

Data are pooled results across four readers (e.g., 2445 × 4 = 9780 lesions). Values are percentages (numerators/denominators) with [95% confidence intervals]. A *p*-value < 0.05 indicates a statistically significant difference between LI-RADS v2018 and KLCA-NCC v2022

*LI-RADS* Liver Imaging Reporting and Data System, *HCC* hepatocellular carcinoma, *KLCA-NCC* Korean Liver Cancer Association-National Cancer Center, *PPV* positive predictive value, *NPV* negative predictive value

**Fig. 3:**
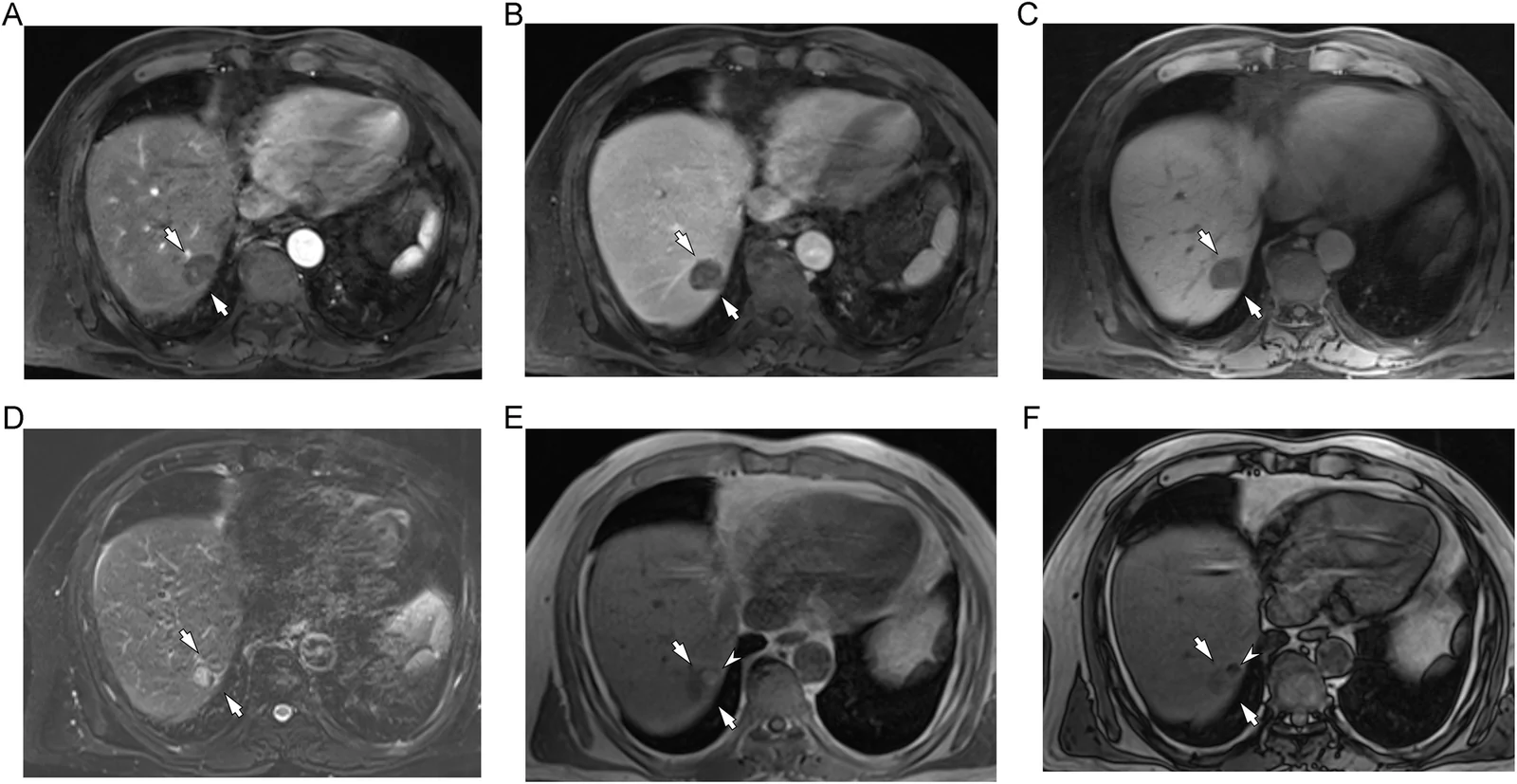
A 57-year-old male patient with hepatitis B-related cirrhosis and surgically confirmed HCC. **A** A 21.5 mm mass (arrows) in segment 7 shows no APHE, **B** non-peripheral portal washout, **C** nontargetoid HBP hypointensity, and **D** mild-moderate T2 hyperintensity. **E** In the in-phase and **F** opposed-phase, gross fat (arrowheads) is observed in the mass. By LI-RADS v2018, the mass is classified as LR-4 based on size and one major feature (portal washout), while AFs (**C**, **D**, **E**, **F**) have a limited role. By KLCA-NCC v2022, the mass does not meet “definite HCC” criteria due to a lack of APHE. Instead, this is classified as “probable HCC” by having at least one AF suggesting malignancy in general (**D**) and at least one HCC-specific AF (**E**, **F**). HCC, hepatocellular carcinoma; APHE, arterial phase hyperenhancement; HBP, hepatobiliary phase; LI-RADS, Liver Imaging Reporting and Data System; KLCA-NCC, Korean Liver Cancer Association-National Cancer Center; AF, ancillary feature

**Fig. 4:**
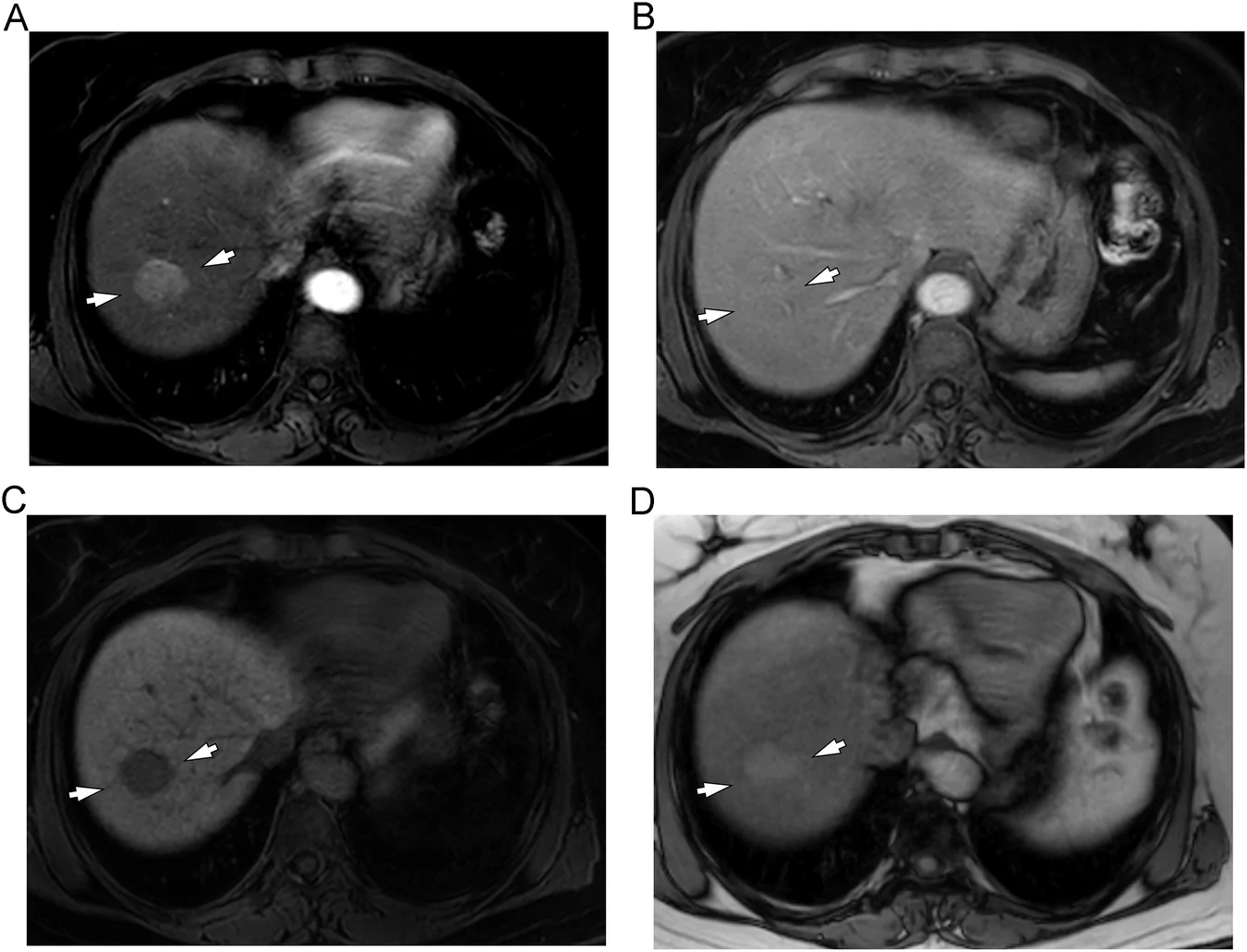
A 66-year-old female patient with metabolic dysfunction-associated steatohepatitis-cirrhosis and surgically confirmed HCC. **A** A 25 mm mass (arrows) in liver segment 8 shows non-rim APHE, **B** no portal washout, **C** nontargetoid HBP hypointensity, and (**D**) fat sparing on opposed-phase. The mass is categorized as LR-4 in LI-RADS v2018 based on size and non-rim APHE (**A**); AFs suggesting malignancy in general (**C**, **D**) are observed, but do not alter the category. The mass is “definite HCC” in KLCA-NCC v2022 based on two major features, non-rim APHE (**A**) and HBP hypointensity (**C**). HCC, hepatocellular carcinoma; APHE, arterial phase hyperenhancement; HBP, hepatobiliary phase; LI-RADS, Liver Imaging Reporting and Data System; KLCA-NCC, Korean Liver Cancer Association-National Cancer Center

*“probable HCC”—*On MRI, the PPV of “LR-4” was comparable to that of KLCA-NCC “probable HCC” (68.2% vs. 64.1%, *p* = 0.141) (Table 3, S5, and Fig. 3). On CT, KLCA-NCC “probable HCC” had a higher PPV than LR-4 (83.8% vs. 73.9%, *p* = 0.012) (Tables 3 and S5). On MRI, 44.9% (582/1295) of LR-4 were KLCA-NCC “definite HCC” (Fig. 4) while 2.7% (16/582) of KLCA-NCC “probable HCC” was LR-5 (Table 4). On CT, 0.5% (2/433) of LR-4 was KLCA-NCC “definite HCC” while 54.5% (54/99) of KLCA-NCC “probable HCC” was LR-5 (Table 4 and Fig. 3).

**Table 3:** Comparison of the performance of LR-4 in LI-RADS and “probable HCC” in KLCA-NCC for diagnosing HCC

Guidelines	PPV (%)			
	All lesions	Presence of non-rim APHE	Absence of non-rim APHE	*p*-value^a^
MRI (*n* = 2445)				
LI-RADS v2018	68.2 (883/1295) [63.7, 72.4]	74.0 (543/734) [68.8, 78.6]	60.6 (340/561) [53.2, 67.5]	0.002
KLCA-NCC v2022	64.1 (373/582) [57.6, 70.1]	42.3 (91/215) [32.1, 53.3]	76.8 (282/367) [69.8, 82.7]	< 0.001
Difference	4.1 [−1.4, 9.6]	31.7 [21.6, 41.7]	−16.2 [−21.9, −10.5]	
* p*-value^b^	0.141	< 0.001	< 0.001	
CT (*n* = 1590)				
LI-RADS v2018	73.9 (320/433) [67.8, 79.2]	68.6 (129/188) [58.6, 77.1]	78.0 (191/245) [70.8, 83.8]	0.102
KLCA-NCC v2022	83.8 (83/99) [75.1, 89.9]	83.8 (83/99) [75.1, 89.9]	N/A^c^	
Difference	−9.9 [−18, −2.2]	−15.2 [−24.5, −5.9]	—	
* p*-value^b^	0.012	0.001	—	

Data are pooled results across four readers (e.g., 2445 × 4 = 9780 lesions). Values are percentages (numerators/denominators) with [95% confidence intervals]

*LI-RADS* Liver Imaging Reporting and Data System, *HCC* hepatocellular carcinoma, *KLCA-NCC* Korean Liver Cancer Association-National Cancer Center, *PPV* positive predictive value, *APHE* arterial phase hyperenhancement

^a, b^ A *p*-value < 0.05 indicates the statistically significant difference ^a^between lesions with and without imaging features or ^b^between LR-4 in LI-RADS v2018 and “probable HCC” in KLCA-NCC v2022

^c^ No focal liver lesions were categorized as “probable HCC”

**Table 4:** Cross-classification between LR-4 in LI-RADS v2018 and “probable HCC” in KLCA-NCC v2022

MRI			
KLCA-NCC v2022 categories in LR-4 observations			
KLCA-NCC v2022 category	All FLLs (*n* = 1295)	Non-HCC (*n* = 412)	HCC (*n* = 883)
“Do not apply criteria”	29 (2.2)	21 (1.6)	8 (0.6)
“Indeterminate nodule”	315 (24.3)	161 (12.4)	154 (11.9)
“Probable HCC”	369 (28.5)	113 (8.7)	256 (19.8)
“Definite HCC”	582 (44.9)	117 (9.0)	465 (35.9)
**LI-RADS v2018 categories in KLCA-NCC “probable HCC” observations**			
**LI-RADS v2018 category**	**All FLLs** (***n*** = **582**)	**Non-HCC** (***n*** = **209**)	**HCC** (***n*** = **373**)
LR-1	0 (0.0)	0 (0.0)	0 (0.0)
LR-2	3 (0.5)	3 (0.5)	0 (0.0)
LR-3	79 (13.6)	66 (11.3)	13 (2.2)
LR-4	369 (63.4)	113 (19.4)	256 (44.0)
LR-5	16 (2.7)	0 (0.0)	16 (2.7)
LR-M	103 (17.7)	25 (4.3)	78 (13.4)
LR-NC	12 (2.1)	2 (0.3)	10 (1.7)
**CT**			
**KLCA-NCC v2022 categories in LR-4 observations**			
**KLCA-NCC v2022 category**	**All FLLs** (***n*** = **433)**	**Non-HCC** (***n*** = **113)**	**HCC** (***n*** = **320)**
“Do not apply criteria”	0 (0.0)	0 (0.0)	0 (0.0)
“Indeterminate nodule”	390 (90.1)	103 (23.8)	287 (66.3)
“Probable HCC”	41 (9.5)	9 (2.1)	32 (7.4)
“Definite HCC”	2 (0.5)	1 (0.2)	1 (0.2)
**LI-RADS v2018 categories in KLCA-NCC “probable HCC” observations**			
**LI-RADS v2018 category**	**All FLLs** (***n*** = **99)**	**Non-HCC** (***n*** = **16**)	**HCC (*n*** = **83)**
LR-1	0 (0.0)	0 (0.0)	0 (0.0)
LR-2	0 (0.0)	0 (0.0)	0 (0.0)
LR-3	0 (0.0)	0 (0.0)	0 (0.0)
LR-4	41 (41.4)	9 (9.1)	32 (32.3)
LR-5	54 (54.5)	6 (6.1)	48 (48.5)
LR-M	4 (4.0)	1 (1.0)	3 (3.0)
LR-NC	0 (0.0)	0 (0.0)	0 (0.0)

Data are pooled results across four readers (e.g., 2445 × 4 = 9780 lesions). Values are numbers (percentage)

*LI-RADS* Liver Imaging Reporting and Data System, *HCC* hepatocellular carcinoma, *KLCA-NCC* Korean Liver Cancer Association-National Cancer Center, *FLL* focal liver lesion

**Fig. 5:**
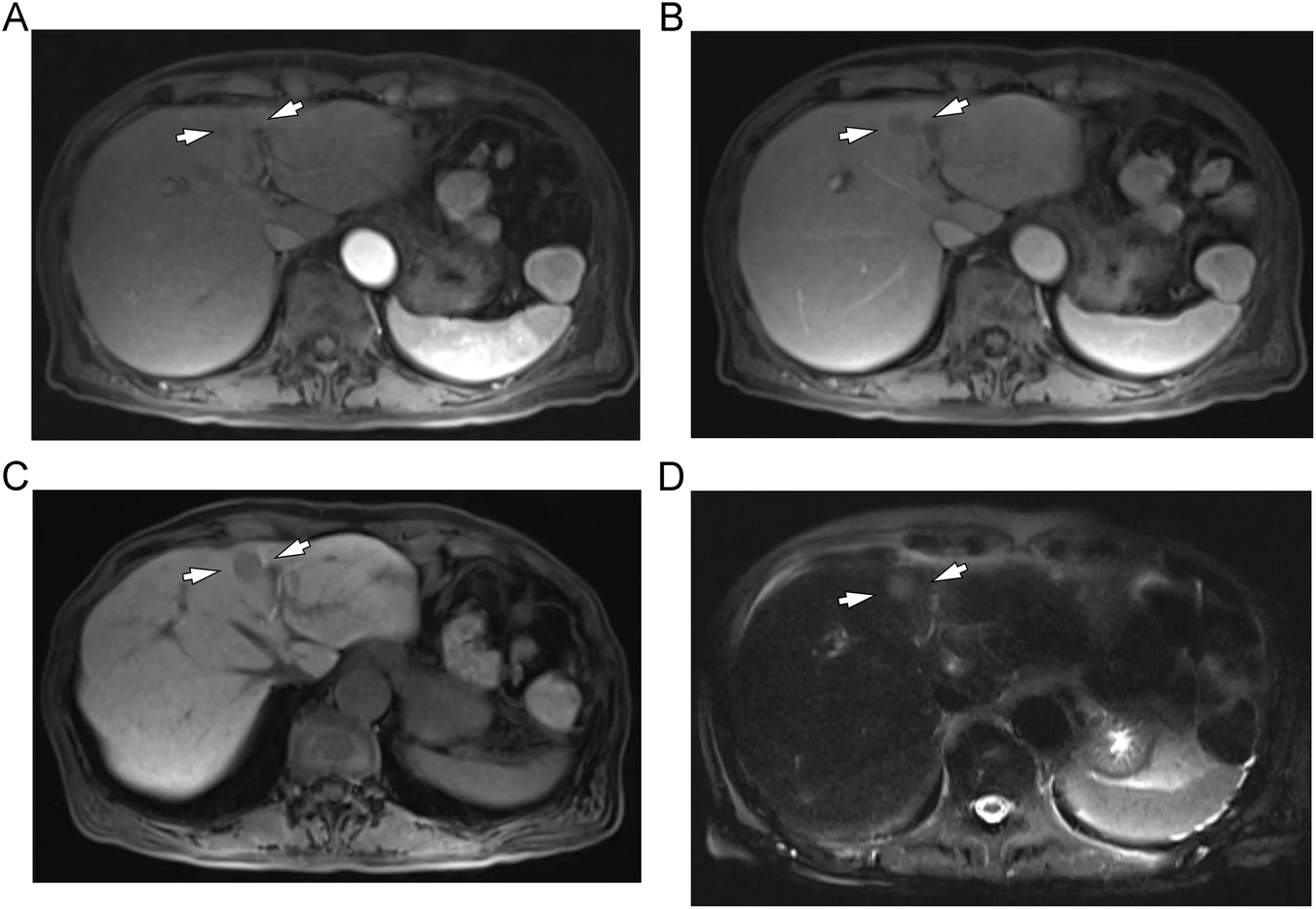
A 87-year-old male patient with chronic hepatitis B and surgically confirmed HCC. **A** A 16.2 mm nodule (arrows) in segment 4 shows no APHE, **B** non-peripheral portal washout, **C** nontargetoid HBP hypointensity and **D** mild-moderate T2 hyperintensity. No HCC-specific AFs are observed. The nodule is classified as LR-4 in LI-RADS v2018 based on the lack of a major feature (**A**) and the presence of AF, suggesting malignancy in general (**C**, **D**). In KLCA-NCC v2022, this is an “indeterminate nodule” because it lacks a major feature (**A**) and HCC-specific AFs, which are required to be “probable HCC” together with AFs suggesting malignancy in general for lesions without APHE. HCC, hepatocellular carcinoma; APHE, arterial phase hyperenhancement; HBP, hepatobiliary phase; AF, ancillary feature; LI-RADS, Liver Imaging Reporting and Data System; KLCA-NCC, Korean Liver Cancer Association-National Cancer Center

*“indeterminate nodule”*—Proportion of HCC was lower in LR-3 compared with KLCA-NCC “indeterminate nodule” on MRI: 13.2% vs. 40.8%, respectively (*p* < 0.001) (Table S6 and Fig. 5). On CT, LR-3 had a higher pooled proportion of HCC than KLCA-NCC “indeterminate nodule”: 52.0% vs. 46.2%, respectively (*p* = 0.001, Table S6).

### PPV of “Probable HCC” category depending on imaging features

*Presence of non-rim APHE*—On MRI, LR-4 with non-rim APHE showed higher PPV than LR-4 without non-rim APHE (74.0% vs. 60.6%, *p* = 0.002). However, no PPV difference was observed on CT (68.6% in LR-4 with non-rim APHE vs. 78.0% in LR-4 without non-rim APHE, *p* = 0.102) (Table 3). In KLCA-NCC, “probable HCC” without non-rim APHE had a higher PPV than “probable HCC” with non-rim APHE on MRI (76.8% vs. 42.3%, *p* < 0.001) (Table 3). No observations without non-rim APHE were classified as “probable HCC” on CT.

**Fig. 6:**
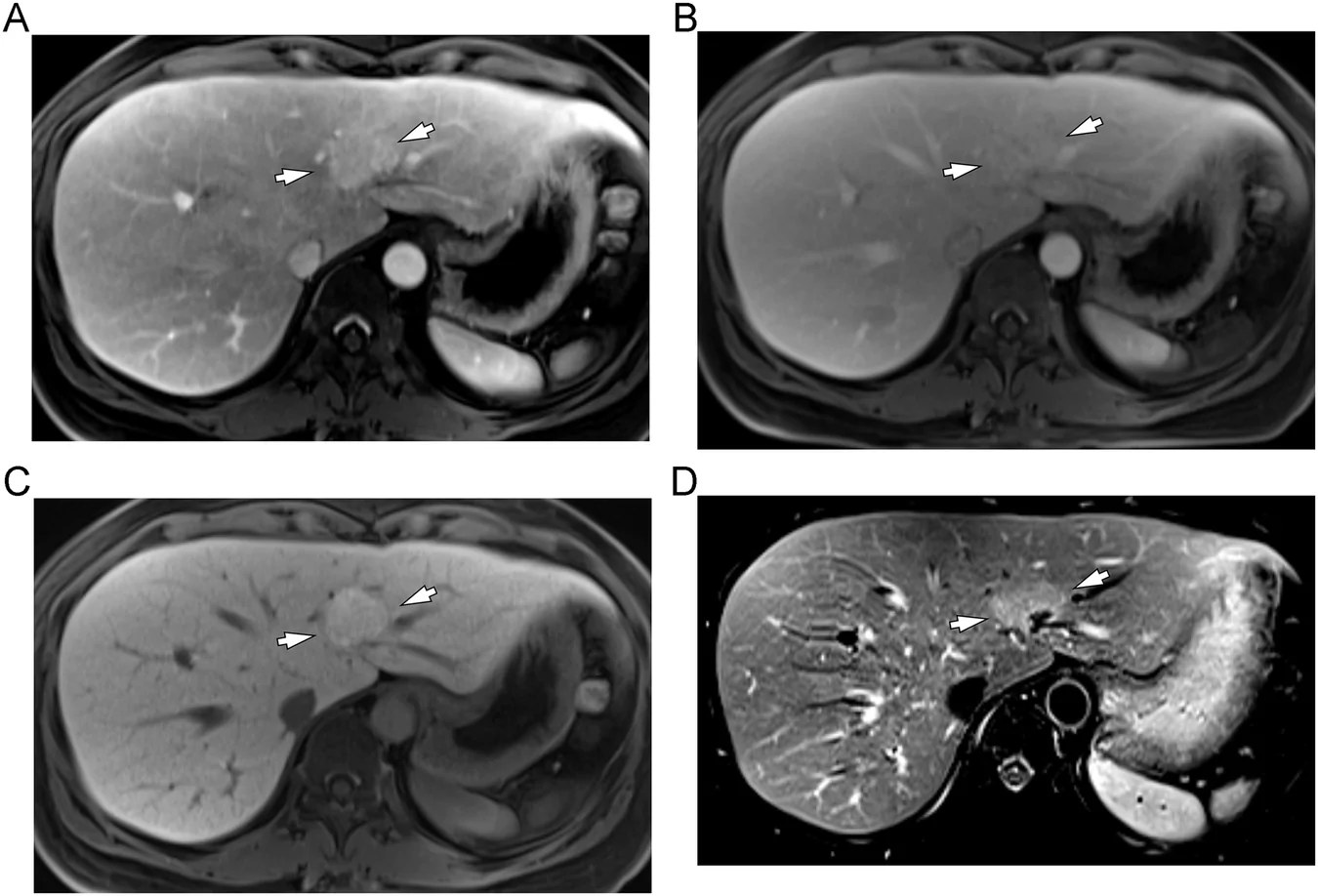
A 50-year-old female patient with chronic hepatitis B and focal nodular hyperplasia on biopsy. **A** A 28.8 mm mass (arrows) in liver segment 3 shows non-rim APHE, **B** no portal washout, **C** HBP hyperintensity without a central scar, and **D** mild-moderate T2 hyperintensity. Both LI-RADS v2018 and KLCA-NCC v2022 categorize the mass as LR-4 and “probable HCC,” respectively, based on size and one major feature (**A**) in LI-RADS; and based on one major feature (**A**) and AF suggesting malignancy in general (**D**) in KLCA-NCC. APHE, arterial phase hyperenhancement; HBP, hepatobiliary phase; HCC, hepatocellular carcinoma; LI-RADS, Liver Imaging Reporting and Data System; KLCA-NCC, Korean Liver Cancer Association-National Cancer Center

*Presence of HCC-specific AFs*—On MRI, LR-4 with HCC-specific AFs showed higher PPV than LR-4 without HCC-specific AFs (80.3% vs. 63.5%, *p* < 0.001); KLCA-NCC “probable HCC” with HCC-specific AFs had also higher PPV than those without HCC-specific AFs (74.9% vs. 28.1%, *p* < 0.001) (Table 5) (Figs. 2 and 6). When the analysis was limited to observations with non-rim APHE, both LR-4 and KLCA-NCC “probable HCC” demonstrated significantly higher PPVs in the presence of HCC-specific AFs than in their absence (*p* = 0.006 and *p* < 0.001, respectively). However, CT did not reveal a difference in PPV between LR-4 with and without HCC-specific AFs (*p* = 0.081) (Table 5).

**Table 5:** Impact of HCC-specific AFs in LR-4 in LI-RADS and “probable HCC” in KLCA-NCC for diagnosing HCC

Guidelines	PPV (%)		*p*-value^a^	PPV (%)		*p*-value^a^
	Presence of HCC-specific AFs	Absence of HCC-specific AFs		Non-rim APHE and HCC-specific AFs	Non-rim APHE without HCC-specific AFs	
MRI (*n* = 2445)						
LI-RADS v2018	80.3 (289/360) [73.8, 85.5]	63.5 (594/935) [58.3, 68.5]	< 0.001	82.3 (144/175) [74.7, 88.0]	71.4 (399/559) [65.4, 76.7]	0.006
KLCA-NCC v2022	74.9 (335/447) [68.6, 80.4]	28.1 (38/135) [18.2, 40.9]	< 0.001	66.3 (53/80) [51.3, 78.5]	28.1 (38/135) [18.2, 40.9]	< 0.001
Difference	5.3 [0.9, 9.8]	35.4 [23.9, 46.9]		16.0 [4.0, 28.1]	43.2 [32.1, 54.4]	
* p*-value^b^	< 0.001	< 0.001		0.009	< 0.001	
CT (*n* = 1590)						
LI-RADS v2018	82.8 (48/58) [69.3, 91.1]	72.5 (272/375) [66.1, 78.1]	0.081	82.4 (14/17) [56.5, 94.4]	67.3 (115/171) [56.9, 76.1]	0.123
KLCA-NCC v2022	83.8 (83/99) [75.1, 89.9]	N/A^c^		83.8 (83/99) [75.1, 89.9]	N/A^c^	
Difference	−1.1 [−12.9, 10.7]	—		−1.5 [−18.2, 15.2]	—	
* p*-value^b^	0.858	—		0.862	—	—

Data are pooled results across four readers (e.g., 2445 × 4 = 9780 lesions). Values are percentages (numerators/denominators) with [95% confidence intervals]

*HCC* hepatocellular carcinoma, *AF* ancillary feature, *LI-RADS* Liver Imaging Reporting and Data System, *KLCA-NCC* Korean Liver Cancer Association-National Cancer Center; *PPV* positive predictive value, *APHE* arterial phase hyperenhancement

^a, b^ A *p*-value < 0.05 indicates the statistically significant difference ^a^between lesions with and without imaging features or ^b^between LR-4 in LI-RADS v2018 and “probable HCC” in KLCA-NCC v2022

^c^ No focal liver lesions were categorized as “probable HCC”

### Per-reader analyses

Per-reader PPV analyses demonstrated consistent findings across the four readers on both MRI and CT. The direction of between-system differences for “definite HCC,” “probable HCC,” and “indeterminate nodule” was preserved in the majority of individual readers, with magnitudes comparable to the pooled estimates (Tables S3–S6).

### Impact of LR-4 observations with non-rim APHE and extended washout timing

When both LR-5 and LR-4 showed non-rim APHE and TP or HBP hypointensity, they were regarded as test positives, and sensitivity increased to 77.7% while decreasing specificity to 84.9% (*p *< 0.001 for both) (Table S7). Sensitivity, specificity, PPV and NPV of LI-RADS with extended washout timing did not show a difference compared with those using “definite HCC” in KLCA-NCC (*p *= 0.08, 0.05, 0.08 and 0.43, respectively).

### Interobserver agreement

On MRI, the interobserver agreement (*κ*) was 0.637 for LR-5, 0.533 for LR-4 and 0.354 for LR-3 (Table S8). In KLCA-NCC, Fleiss kappa was 0.653, 0.425 and 0.608 for “definite HCC”, “probable HCC,” and “indeterminate nodule” respectively.

Fleiss kappa for LR-5, LR-4 and LR-3 was 0.252 to 0.654 on CT (Table S8). Those for “definite HCC”, “probable HCC,” and “indeterminate nodule” of KLCA-NCC v2022 were 0.643, 0.032 and 0.650, respectively, while Gwet’s AC1 was 0.770, 0.970, and 0.777, respectively (Table S8).

## Discussion

Our study compared conceptually corresponding diagnostic categories in two probability-based diagnostic guidelines for HCC. On MRI, the proportion of HCC was 93.9% for LR-5 and 91.7% for KLCA-NCC “definite HCC,” but declined to 68.2% and 64.1% in LR-4 and KLCA-NCC “probable HCC,” and further decreased in LR-3 and KLCA-NCC “indeterminate nodule”. On CT, a similar stepwise decline in HCC proportion was observed across categories. This demonstrates the effectiveness of current noninvasive probability-based diagnostic criteria.

In a head-to-head comparison, LR-5 showed lower sensitivity but higher specificity and PPV compared with KLCA-NCC “definite HCC” on MRI, which confirmed prior studies reporting high specificity by accepting only portal washout on gadoxetic acid-enhanced MRI in LI-RADS [14–16]. However, LR-4 revealed lower PPV than KLCA-NCC “probable HCC”, despite statistical insignificance. When both “definite HCC” and “probable HCC” were considered test positives, we observed no significant differences in all diagnostic metrics between the two. This alignment in diagnostic performance suggests that LR-4 represents a more inclusive diagnostic category compared to KLCA-NCC “probable HCC”. Notably, when LI-RADS adopted extended washout timing by including LR-4 observations with non-rim APHE and TP or HBP defect as test positive, the diagnostic performance became comparable to that of KLCA-NCC “definite HCC”, with acceptable PPV (92.1%). This suggests that washout timing differences primarily account for the “definite HCC” performance gap, while the impact of other features—such as EC as a major feature vs. AF and nontargetoid LR-M features—may be limited. Given the ongoing discussions regarding the extension of washout timing for gadoxetic acid-enhanced MRI [17], these findings may provide useful empirical evidence on the sensitivity-specificity trade-off underlying such considerations, though the optimal balance likely depends on regional HCC prevalence and preferred treatment options.

In addition to the inclusiveness of “definite HCC” criteria, another key factor that causes differences between the two systems is their unique approaches to AFs. Cross-tabulation revealed that 63.4% of KLCA-NCC “probable HCC” were LR-4, while only 28.5% of LR-4 were “probable HCC” in KLCA-NCC (44.9% as “definite HCC”, 24.3% as “indeterminate nodules”). This confirms that these two are not interchangeable, reflecting KLCA-NCC’s stricter criteria requiring both malignancy-suggestive and HCC-specific AFs for lesions lacking APHE, fewer AFs and no benignity-suggesting AFs. In contrast, LI-RADS takes a more inclusive stance on AFs, allowing LR-4 based on various combinations of major features and AFs. Although LI-RADS uses nontargetoid LR-M features to maintain specificity in non-“LR-5” observations, this systematic difference accounts for why LR-4 includes a broader range of lesions (*n* = 1295) compared to “probable HCC” in KLCA-NCC (*n* = 582). This pattern demonstrates that LR-4 encompasses a more heterogeneous group of lesions compared to “probable HCC” in the KLCA-NCC system.

The moderate PPV of “probable HCC” in both systems supports the current recommendations. KLCA-NCC advises either a repeated imaging follow-up in three months or a biopsy, with occasional treatment plans through a multidisciplinary team discussion [5]. Similarly, LI-RADS recommends multidisciplinary team discussions for LR-4, which may include imaging, biopsy, and occasionally immediate treatment without a biopsy [18]. Given these modest PPVs, prioritizing repeated imaging and biopsy over immediate treatment appears reasonable. However, the current recommendations encompass diverse management options without clear guidance. This ambiguity partly stems from the inherent heterogeneity within these categories. Identifying imaging features that drive this variability could enable more refined risk stratification and inform individualized management decisions.

In this context, we found that the PPV of “probable HCC” varied based on imaging features. LR-4 with non-rim APHE demonstrated higher PPV compared to those without (74.0% vs. 60.6%), suggesting that lesions with non-rim APHE may have a higher probability of HCC. Interestingly, KLCA-NCC showed an opposite pattern, where “probable HCC” with non-rim APHE had lower PPV than those without (42.3% vs. 76.8%), reflecting the stricter criteria for lesions without APHE (both malignancy-suggestive and HCC-specific AFs). Indeed, “probable HCC” with HCC-specific AFs showed higher PPV compared to those without in both guidelines. These findings suggest non-rim APHE and HCC-specific AFs could serve as subcategorization criteria, enabling more refined probability-based stratification. We may consider treatment for LR-4 with HCC-specific AFs (PPV 80.3%), short-term follow-up or biopsy for LR-4 without (PPV 64%), and imaging surveillance for KLCA-NCC “probable HCC” lacking both features (PPV 28%). While a meta-analysis found upgrading LR-4 to LR-5 using AFs did not improve diagnostic performance [19], our findings demonstrate their value for within-category stratification, supporting AF-based subcategorization in future revisions.

We also observed a notable difference in the lowest probability categories. On MRI, the HCC proportion in LR-3 (13.2%) was substantially lower than that of KLCA-NCC “indeterminate nodule” (40.8%), despite both representing the lowest HCC likelihood. In LI-RADS, benignity-favoring AFs downgrade to LR-2, while malignancy-favoring AFs upgrade to LR-4, filtering both low- and high-risk lesions from LR-3. In contrast, KLCA-NCC does not incorporate benignity-suggesting AFs and has stricter criteria for “probable HCC,” resulting in a broader spectrum of lesions remaining in this category. This difference highlights how the systematic use of AFs contributes to more refined risk stratification in the intermediate categories.

Performance of each category is modality dependent. Unlike MRI, LR-4 had lower PPV than KLCA-NCC “probable HCC” (73.9% vs. 83.8%, *p* = 0.012) on CT, reflecting modality-dependent performance difference. Notably, the “probable HCC” category was rarely assigned in KLCA-NCC on CT (*n* = 99) compared to MRI (*n* = 582), as the absence of MRI-specific AFs, such as mild-moderate T2 hyperintensity and restricted diffusion, substantially limits classification options. Furthermore, more than half of the KLCA-NCC “probable HCC” lesions on CT (54.5%) were classified as LR-5 on CT, in contrast to only 2.7% on MRI. This discrepancy arises from the different roles of EC in two guidelines: EC is an AF in KLCA-NCC, while it is a major feature in LI-RADS. This aligns with our observations that LR-5 showed higher sensitivity than “definite HCC” in KLCA-NCC on CT, unlike MRI. Additionally, HCC-specific AFs did not significantly improve PPV in LR-4 on CT (82.8% with vs. 72.5% without), unlike MRI. This may reflect a higher baseline PPV of LR-4 without AFs on CT (72.5% vs. 63.5% on MRI). Lower CT contrast resolution for washout may have shifted some HCCs from LR-5 to LR-4. On the other hand, the proportion of HCC was 52.0% in LR-3 on CT, higher than that of KLCA-NCC “indeterminate nodule”. This paradoxical reversal likely confirms prior meta-analysis [20, 21] and may reflect several modality-specific factors. First, AF-based upgrade from LR-3 to LR-4 is limited on CT, as CT-assessable AFs such as corona enhancement, fat sparing in solid mass, and other HCC-specific AFs are infrequent compared with MR-specific AFs [19]. This recognized CT AF deficit has motivated active investigation of new CT-specific AFs, such as precontrast low attenuation [22]. Second, the lower sensitivity of CT for diagnosing LR-5 compared with MRI [23] may shift a fraction of HCCs that fulfill LR-5 criteria on MRI down to LR-4 or LR-3 on CT, which is a modality-level limitation distinct from AF availability. The relatively high baseline PPV of LR-4 without HCC-specific AFs on CT (72.5%, vs. 63.5% on MRI) indirectly supports this downward trend. Third, an asymmetric benign-filtering effect operates between the two systems on CT: LI-RADS provides an LR-2 downgrade pathway via benignity-suggesting AFs, whereas KLCA-NCC v2022 lacks an equivalent mechanism. Concordantly, the KLCA-NCC “indeterminate nodule” pool on CT (*n* = 1681) was substantially larger than LR-3 (*n* = 790), potentially diluting HCC proportion by retained benign lesions while LR-3 was concurrently enriched in HCCs.

Clinically, the implication is direct. The HCC probability of CT LR-3 ( ~ 52%) approaches that of CT LR-4 (73.9%) and substantially exceeds that of MRI LR-3 (13.2%). The current LI-RADS recommendation of imaging surveillance for LR-3, based on its low HCC probability, may not be appropriate when LR-3 is assigned on CT, in which case MRI characterization should be considered. This further supports the need to refine the intermediate categories, CT-specifically.

Further, the rarity of KLCA-NCC “probable HCC” on CT is also reflected in the interobserver agreement. Fleiss kappa for this category was extremely low (0.032), while Gwet’s AC1 was 0.970. This discrepancy represents a well-recognized kappa paradox, in which rarely assigned categories yield paradoxically low kappa values despite high actual agreement [12]. This indicates that the four readers consistently agreed that observations did not meet the “probable HCC” criteria on CT, further underscoring the limited applicability of this category to CT. These findings suggest current “probable HCC” criteria, developed primarily for MRI, may require modality-specific refinement for CT.

Our study had several limitations. First, the retrospective design introduced inevitable bias. Second, we mandatorily applied AFs with tie-breaking rules, whereas the current LI-RADS recommendation leaves AF application as optional. This may have shifted some observations between the LR-3 and LR-4 categories. However, LI-RADS rules do not permit AF-based upgrades to LR-5, so the high-certainty category comparisons are unaffected. Nevertheless, studies found that AF application did not significantly change the proportion of HCCs in each LI-RADS category, suggesting a limited impact on category-level PPV [24, 25]. Moreover, our mandatory approach ensured symmetric AF application to both classification systems and may reduce inter-reader variability from inconsistent AF use, which has been recognized as a known limitation of optional application [17, 24]. Third, we included non-cirrhotic hepatitis C patients, although it is not a target population of LI-RADS v2018. However, a recent study revealed the feasibility of LI-RADS in this population [26]. Fourth, incorporation bias may exist as malignant lesions required pathologic confirmation, whereas benign lesions were diagnosed by histology or clinical criteria, in line with the known impact of reference standard on LI-RADS performance [27]. To mitigate the concerns, clinically diagnosed benign lesions required both composite imaging (contrast-enhanced ultrasound, CT, MRI) with ≥2-year stability. Fifth, we did not include chronological AFs because they were unavailable for most patients. Sixth, although KLCA-NCC v2022 was released after our data collection period (2015–2018), gadoxetic acid-enhanced MRI protocols including TP (3 min) and HBP (20 min) were well established before the study period, and retrospective application of updated criteria to both systems under identical conditions is unlikely to introduce differential bias. Seventh, it should be noted that management recommendations for “probable HCC” may differ between Asian and Western clinical contexts, as local pre-test probability and preferred treatment option may modulate how the proposed PPV-based stratification is operationalized. Lastly, the predominantly hepatitis B virus-cirrhotic population may limit generalizability to hepatitis C and metabolic dysfunction-associated steatohepatitis (MASLD) dominant western populations, as the prevalence of major and AFs may differ across etiologies [28], although the effect of background steatosis on washout remains inconclusive [29, 30]. However, fat sparing in solid mass did not reach statistical significance in a recent IPD meta-analysis [31]. And fat in mass, despite its association with steatohepatitic HCC [32], is not etiology-specific, occurring across viral hepatitis and non-steatotic livers [33]. Thus, the impact of differing AF profiles on the current comparison is likely limited, though dedicated validation in MASLD-predominant cohorts is warranted.

In conclusion, both LI-RADS v2018 and KLCA-NCC v2022 demonstrated effective probability-based risk stratification within their respective categories. However, different PPV of conceptually corresponding categories suggests they are not interchangeable between the systems. On CT, category performance differed from MRI due to the unavailability of MRI-specific AFs, highlighting the need for modality-specific refinement.

## Supplementary information

ELECTRONIC SUPPLEMENTARY MATERIAL

## Data Availability

Individual patient data will not be publicly shared due to privacy concerns. Data generated or analyzed during the study are available from the corresponding author upon request.

## Abbreviations

AF: Ancillary feature
APHE: Arterial phase hyperenhancement
EC: Enhancing capsule
FLL: Focal liver lesion
HCC: Hepatocellular carcinoma
KLCA-NCC: Korean Liver Cancer Association-National Cancer Center
LI-RADS: Liver Imaging Reporting and Data System
PPV: Positive predictive value
