# Supplementary material for: LI-RADS v2018 versus KLCA-NCC v2022: comparison of probability-based HCC categories
Source: Eur Radiol. 2026 Jun 26;36(11):8451–64. doi: 10.1007/s00330-026-12696-0 (PMC13661650; doi:10.1007/s00330-026-12696-0)
Supplement: Supplementary file 1 [file 330_2026_12696_MOESM1_ESM.docx]

**SUPPLEMENT**

**SUPPLEMENTARY MATERIALS AND METHODS**

**MRI examination**

Detailed information was published elsewhere [1]. MRI examinations were performed using field of view of 350 x 350 to 380 x 380 mm. Regarding T1-weighted sequences (precontrast, arterial, portal venous, transitional and hepatobiliary phases), a three-dimensional (3D) gradient echo sequence was used with the following scan parameters: repetition time (TR)/echo time (TE), 2.8–3.5/1.3–2.1 msec; flip angle 9–11°; matrix 256 x 280 to 384 x 230, slice thickness 3–4 mm. Diffusion-weighted imaging was obtained using at least two b-values between 0 and 1000 sec/mm^2^ (a combination of two or three values among 0, 50, 400, 500, 800, and 1000 sec/mm^2^). Heavily T2-weighted imaging was obtained with single-shot spin echo sequence with long TE (160–180 msec) without fat suppression. Fat-suppressed T2-weighted image was obtained using spin echo sequence in either multiple breath-hold or respiratory gating: TR/TE, 80–100 msec; slice thickness 5–6 mm.

**CT examination**

Detailed scan information was described elsewhere [2]. CT examinations were performed at the multi-detector scanners with 16–256 channels. After pre-contrast phase acquisition, an iodine contrast agent (350–375 mgI/mg) was administered intravenously at a dose of 1.5–1.6 mL/kg, with an infusion rate of 3–5 mL/s, followed by a saline flush of 20–40 mL using an automatic power injector. The peak voltage was 80–140 kVp depending on the patients’ body habitus and institutional settings, and vendor-provided dose modulation was applied. Arterial phase was obtained using a bolus tracking technique, with the 10–15 sec delay initiated upon reaching a threshold enhancement of 100–150 HU in the descending thoracic aorta. Portal venous phase and delayed phase were obtained at 55–70 sec and 3 min after contrast media administration. Images were reconstructed using filtered back projection, vendor-provided hybrid or model-based iterative reconstruction algorithms available at the scanners.

**Web platform-based templates and automatic diagnostic algorithms**

In Liver Imaging Reporting and Data System (LI-RADS) v2018, Ancillary features (AFs) suggesting malignancy include a) mild-moderate T2 hyperintensity; b) restricted diffusion; c) corona enhancement; d) fat or iron sparing in solid mass; e) transitional phase (TP) or hepatobiliary phase (HBP) hypointensity; f) subthreshold growth; g) ultrasound visibility as discrete nodule. In addition, hepatocellular carcinoma (HCC)-specific AFs include a) nonenhancing capsule; b) nodule-in-nodule appearance; c) mosaic appearance; and d) blood products or fat in the mass [3]. In Korean Liver Cancer Association-National Cancer Center (KLCA-NCC) v2022, AFs suggesting malignancy include a) mild-moderate T2 hyperintensity; b) restricted diffusion and c) threshold growth [4]. And HCC-specific AFs in KLCA-NCC include a) enhancing or nonenhancing capsule; b) nodule-in-nodule appearance; c) mosaic architecture; and d) blood products or fat in the mass [4]. We did not include threshold/subthreshold growth and ultrasound visibility as discrete nodule in the review due to absence of applicable review items. According to KLCA-NCC v2022, at both AF suggesting malignancy and HCC-specific AF are needed to be “probable HCC” in hepatic observations without arterial phase hyperenhancement (APHE) while at least one AF is needed in observations with APHE, regardless of the category. In LI-RADS, AFs suggesting benignity include a) parallels blood pool enhancement; b) undistorted vessels; c) iron in mass, more than liver; d) marked T2 hyperintensity; e) HBP isointensity. Among the AFs suggesting benignity, size stability ≥ 2 years or size reduction were not included in the review since follow-up image is not included in a review session.

LI-RADS category was assigned based on the criteria [3]. KLCA-NCC v2022 was assigned as follows: Observation without targetoid features and features suggesting hemangiomas (marked T2 hyperintensity) were assigned to “not applying criteria”. In remaining observations, lesions with non-rim APHE and nonperipheral washout, TP or HBP hypointensity were assigned to “definite HCC” category. In observations not meeting the criteria of “definite HCC”, “probable HCC” was assigned when non-rim APHE and either AFs suggesting malignancy or HCC-specific were observed; AFs of both categories (malignancy and HCC-specific) were required to be “probable HCC” if non-rim APHE was absent. The remaining lesions were categorized as “indeterminate nodule” [4].

**Imaging criteria of clinically diangnosed benign lesions**

*Hemangiomas —* Hemangiomas were clinically diagnosed based on marked hyperintensity on T2-weighted image (T2WI), slow and discontinuous peripheral nodular enhancement pattern on contrast-enhanced CT or dynamic MRI as well as no clinically significant interval change during follow-up [5]. Flash-filling hemangiomas are defined as observations with marked hyperintensity on T2WI and APHE and stable on follow-up [6].

*FNH or FNH-like nodules —* Clinical diagnosis of FNH or FNH-like nodules was made based on following imaging features: radial spoke-wheel centrifugal APHE and hepatobiliary phase iso to hyperintensity with central scar which remained stable on follow-up images [7]. If the background liver is cirrhotic, then the observations are defined as FNH-like nodules [8].

*Focal eosinophilic liver disease —* Clinical diagnosis of focal eosinophilic liver disease was made based on following imaging features and clinical course: serum eosinophilia, ill-defined margin, non-rim APHE, HBP defect, size discrepancy between HBP and T2-weighted image [9, 10]. On follow-up, the observations regressed with resolved eosinophilia.

*Vascular malformation —*10 mm observation with APHE and HBP defect was seen, which also showed similar enhancement patterns to hepatic vessels. The observation had venous flow on Doppler ultrasound, and it was clinically diagnosed as aneurysmal dilatation of portal vein branch.

**SUPPLEMENTARY RESULTS**

**Study Sample**

A total of 2237 patients were included from 11 tertiary hospitals: 657 patients from Seoul National University Hospital, 273 patients from Samsung Medical Center, 270 patients from Korea University Guro Hospital, 244 patients from Seoul National University Bundang Hospital, 200 patients from Seoul Saint Mary’s Hospital, 198 patients from Korea University Anam Hospital, 125 patients from Severance Hospital, 90 patients from Kon-Kuk University Medical Center, 75 patients from Chung-Ang University Hospital, 55 patients from Gangnam Severance Hospital and 50 patients from Kyung-Hee University Hospital [1].

In CT subgroup, 1455 patients were included: 547 patients from Seoul National University Hospital, 198 patients from Samsung Medical center, 145 patients from Korea University Guro Hospital, 119 patients from Seoul St. Mary’s Hospital, 104 patients from Seoul National University Bundang Hospital, 84 patients from Korea University Anam Hospital, 83 patients from Severance Hospital, 58 patients from Chung-Ang University Hospital, 49 patients from Kon-Kuk University Medical Center, 40 patients from Gangnam Severance Hospital and 28 patients from Kyung-Hee University Hospital [2].

**Focal liver lesions in study sample**

*All patients—* The diagnosis of focal liver lesions (FLLs) were described elsewhere [1]. Approximately 69.3% (1694/2445) of FLLs were HCC followed by benign FLLs (15.8%, 387/2445) non-HCC malignancies (14.9%, 364/2445). Among the non-HCC malignancies (n = 364), cholangiocarcinoma (CCA) is the most common disease entity (n = 222), followed by metastases (n = 60), combined HCC-CCA (cHCC-CCA, n = 55), sarcoma (n = 9), lymphoma (n = 5), poorly or undifferentiated carcinoma (n = 5), neuroendocrine tumor (NET)/ neuroendocrine carcinoma (NEC)/ mixed neuroendocrine non-neuroendocrine neoplasms (MiNEN) (n = 3), epithelioid hemangioendothelioma (n = 2), intermediate carcinoma (n = 2) and squamous carcinoma (n = 1). Regarding the benign FLLs (n = 387), hemangioma is the most common benign tumors (n = 238), then focal nodular hyperplasia (FNH) or FNH-like nodules are the most common (n = 54), followed by cirrhotic nodules (regenerative nodules [RN, n = 14] and dysplastic nodules [DN, n = 24]), focal eosinophilic liver disease (n= 13), angiomyolipoma (AML, n = 10), chronic inflammation (n = 9), necrotic nodules (n = 6), abscess (n = 5), lymphoid hyperplasia (n = 3), focal fat or fat necrosis (n = 3), hepatocellular adenoma (HCA, n = 3), confluent fibrosis (n = 1), IgG4-related sclerosing disease (n = 1), splenosis (n = 1), vascular malformation (n= 1) and Von-Meyenburg complex (n= 1).

In small FLLs (< 20 mm), malignancy was found in 66.7% (511/766), including HCCs (59.1%, 453/766), metastases (4%, 31/766), CCA (2%, 15/766), cHCC-CCA (1.2%, 9/766), lymphoma (0.1%, 1/766), epithelioid hemangioendothelioma (0.1%, 1/766), intermediate carcinoma (0.1%, 1/766). Benign FLLs (33.3%, 255/766) were hemangiomas (22.1%, 169/766), cirrhotic nodules (4.0%, 31/766; RN [n = 11], DN [n= 20]), FNH or FNH-like nodules (3.3%, 25/766), focal eosinophilic liver disease (1.3%, 10/766), necrotic nodules (0.8%, 5/766), AML (0.4%, 3/766), chronic inflammation (0.4%, 3/766), focal fat or fat necrosis (0.4%, 3/766), lymphoid hyperplasia (0.4%, 3/766), HCA (0.3%, 2/766), vascular malformation (0.1%, 1/766).

*CT subgroup analysis—* The diagnoses of FLLs in the CT subgroup was published elsewhere [2]. Among 1590 FLLs, 72.9% (1159/1590) were HCC. Benign FLLs were found in 14.9% (237/1590) and non-HCC malignancies accounted for 12.2% (194/1590). Non-HCC malignancies included CCA (n = 119), combined HCC-CCA (n = 32), metastases (n = 28), sarcoma (n = 6), poorly or undifferentiated carcinoma (n = 3), intermediate carcinoma (n = 2), lymphoma (n = 2), neuroendocrine tumor (n = 1), and EHE (n = 1). Benign FLLs include hemangiomas (n = 149), FNH or FNH-like nodules (n = 31), cirrhotic nodules (RN [n = 10] and DN [n = 13]), AML (n = 8), focal eosinophilic liver disease (n= 8), chronic inflammation (n = 7), abscess (n = 3), focal fat or fat necrosis (n = 3), lymphoid hyperplasia (n = 2), necrotic nodules (n = 2), and Von-Meyenburg complex (n= 1).

In small FLLs (< 20 mm), HCC was 62.8% (334/532). Non-HCC malignancy was found in 6.6% (35/532), and benign FLLs were 30.6% (163/532). Non-HCC malignancies (n = 35) included CCA (n = 12), metastasis (n = 15), combined HCC-CCA (n = 5), lymphoma (n = 1), EHE (n = 1) and intermediate carcinoma (n = 1). Benign FLLs (n = 163) included hemangioma (n = 105), FNH or FNH-like nodules (n = 20), cirrhotic nodules (RN [n = 8], DN [n= 12]), focal eosinophilic liver disease (n = 6), AML (n = 3), focal fat or fat necrosis (n = 3), chronic inflammation (n = 2), lymphoid hyperplasia (n = 2), necrotic nodules (n = 2).

**Clinical diagnosis of benign FLLs**

Clinical diagnoses were made in 192 FLLs in 172 patients: 89.1% (171/192) were hemangiomas and 9.4% were FNH or FNH-like nodules (18/192). The remaining three FLLs were focal eosinophilic liver disease (n = 2) and vascular malformation (n = 1). This was described in the prior studies [1, 2].

**Table S1. Demographic characteristics of the patients in CT subgroup**

| **Variables** | **Values** |
| --- | --- |
| Sex (male: female) | 1101:354 |
| Age (years)  Male  Female | 59 ± 11 (19–88)  59 ± 10 (19–88)  60 ± 12 (20–86) |
| Underlying liver disease  Chronic hepatitis B  Non-viral cirrhosis*  Chronic hepatitis C  Co-infection of hepatitis B and C viruses  Alcoholic and chronic hepatitis B | 1136 (78.1)  222 (15.3)  79 (5.4)  11 (0.8)  7 (0.5) |
| Child-Pugh classification  Chronic liver disease  Class A  Class B  Class C | 620 (42.6)  746 (51.3)  73 (5.0)  16 (1.1) |
| Diagnostic confirmation  Hepatic resection  Biopsy  Clinical follow-up | 1120 (77.0)  240 (16.5)  95 (6.5) |
| Number of FLLs per patient  1  2  3 | 1332 (91.5)  111 (7.6)  12 (0.8) |
| Size of FLLs (n = 1590 lesions)  HCC (n = 1159)  Non-HCC malignancy (n = 194)  Benign (n = 237) | 27.5 mm (18.7–44.4)  42.9 mm (23.1–68.9)  16.1 mm (12.7–23.8) |

Note—: Values are presented as mean ± standard deviation (range), median (IQR) or number (percentage). FLL = focal liver lesion; HCC = hepatocellular carcinoma; *cryptogenic (n = 119), alcoholic (n = 53), metabolic dysfunction associated steatohepatitis (n = 35), biliary cirrhosis (n = 12), Wilson disease (n = 2), autoimmune hepatitis (n = 1).

**Table S2. Comparison between the diagnostic performance of “definite HCC” and “probable HCC” in LI-RADS v2018 and KLCA-NCC v2022**

| **Guidelines** | **Sensitivity (%)** | **Specificity (%)** | **PPV (%)** | **NPV (%)** |
| --- | --- | --- | --- | --- |
| MRI (n = 2445) | | | | |
| LI-RADS v2018 | 83.6 (5663/6776) [82.3, 84.8] | 75.9 (2281/3004) [73.3, 78.4] | 88.7 (5663/6386) [87.2, 90.0] | 67.2 (2281/3394) [64.5, 69.8] |
| KLCA-NCC v2022 | 83.7 (5670/6776) [82.4, 84.9] | 77.1 (2316/3004) [74.6, 79.4] | 89.2 (5670/6358) [87.8, 90.4] | 67.7 (2316/3422) [64.9, 70.3] |
| Difference | -0.1 [-0.8, 0.6] | -1.2 [-3.0, 0.7] | -0.5 [-1.3, 0.3] | -0.5 [-1.6, 0.6] |
| *P*-value | 0.779 | 0.215 | 0.206 | 0.399 |
| CT (n = 1590) | | | | |
| LI-RADS v2018 | 81.6 (3783/4636) [79.8, 83.3] | 78.0 (1345/1724) [74.7, 81.0] | 90.9 (3783/4162) [89.2, 92.3] | 61.2 (1345/2198) [57.5, 64.8] |
| KLCA-NCC v2022 | 75.5 (3499/4636) [73.4, 77.4] | 84.0 (1448/1724) [81.0, 86.6] | 92.7 (3499/3776) [91.1, 94.0] | 56.0 (1448/2584) [52.4, 59.5] |
| Difference | 6.1 [5.2, 7.1] | -5.9 [-7.4, -4.5] | -1.8 [-2.4, -1.2] | 5.2 [4.0, 6.3] |
| *P*-value | <0.001 | <0.001 | <0.001 | <0.001 |

Note—: Data are pooled results across four readers (e.g., 2445 × 4 = 9780 lesions). Values are percentages (numerators/denominators) with [95% confidence intervals]. A *P*-value <0.05 indicates the statistically significant difference between LI-RADS v2018 and KLCA-NCC v2022. HCC = hepatocellular carcinoma; LI-RADS = Liver Imaging Reporting and Data System; KLCA-NCC = Korean Liver Cancer Association-National Cancer Center; PPV = positive predictive value; NPV = negative predictive value.

**Table S3. Performance of “definite HCC” and “probable HCC” in LI-RADS v2018 and KLCA-NCC v2022 in each reader**

| **Guidelines** | **Readers** | **Sensitivity (%)** | **Specificity (%)** | **PPV (%)** | **NPV (%)** |
| --- | --- | --- | --- | --- | --- |
| MRI (n = 2445) | | | | | |
| LI-RADS v2018 | Reader 1 | 86.6 (1467/1694) [84.9, 88.2] | 79.5 (597/751) [76.4, 82.2] | 90.5 (1467/1621) [89.0, 91.8] | 72.5 (597/824) [69.3, 75.4] |
|  | Reader 2 | 84.3 (1428/1694) [82.5, 86.0] | 76.0 (571/751) [72.8, 79.0] | 88.8 (1428/1608) [87.2, 90.3] | 68.2 (571/837) [64.9, 71.4] |
|  | Reader 3 | 70.6 (1196/1694) [68.4, 72.7] | 78.4 (589/751) [75.3, 81.3] | 88.1 (1196/1358) [86.2, 89.7] | 54.2 (589/1087) [51.1, 57.2] |
|  | Reader 4 | 92.8 (1572/1694) [91.4, 94.0] | 69.8 (524/751) [66.3, 73.0] | 87.4 (1572/1799) [85.7, 88.9] | 81.1 (524/646) [77.8, 84.0] |
| KLCA-NCC v2022 | Reader 1 | 89.2 (1511/1694) [87.6, 90.6] | 80.8 (607/751) [77.8, 83.5] | 91.3 (1511/1655) [89.8, 92.6] | 76.8 (607/790) [73.7, 79.7] |
|  | Reader 2 | 81.6 (1382/1694) [79.6, 83.4] | 78.0 (586/751) [74.9, 80.9] | 89.3 (1382/1547) [87.7, 90.8] | 65.3 (586/898) [62.0, 68.4] |
|  | Reader 3 | 70.8 (1199/1694) [68.6, 72.9] | 81.5 (612/751) [78.5, 84.1] | 89.6 (1199/1338) [87.8, 91.1] | 55.3 (612/1107) [52.3, 58.2] |
|  | Reader 4 | 93.2 (1578/1694) [91.8, 94.3] | 68.0 (511/751) [64.6, 71.3] | 86.8 (1578/1818) [85.1, 88.3] | 81.5 (511/627) [78.2, 84.4] |
| CT (n = 1590) | | | | | |
| LI-RADS v2018 | Reader 1 | 83.9 (972/1159) [81.6, 85.9] | 79.8 (344/431) [75.6, 83.5] | 91.8 (972/1059) [89.9, 93.3] | 64.8 (344/531) [60.5, 68.8] |
|  | Reader 2 | 76.1 (882/1159) [73.5, 78.5] | 83.5 (360/431) [79.7, 86.8] | 92.5 (882/953) [90.7, 94.1] | 56.5 (360/637) [52.5, 60.4] |
|  | Reader 3 | 73.8 (855/1159) [71.2, 76.2] | 82.6 (356/431) [78.6, 86.0] | 91.9 (855/930) [89.9, 93.6] | 53.9 (356/660) [50.0, 57.8] |
|  | Reader 4 | 92.7 (1074/1159) [91.0, 94.1] | 66.1 (285/431) [61.5, 70.4] | 88.0 (1074/1220) [86.1, 89.8] | 77.0 (285/370) [72.3, 81.1] |
| KLCA-NCC v2022 | Reader 1 | 78.5 (910/1159) [76.0, 80.8] | 85.4 (368/431) [81.7, 88.5] | 93.5 (910/973) [91.8, 94.9] | 59.6 (368/617) [55.6, 63.6] |
|  | Reader 2 | 71.0 (823/1159) [68.3, 73.6] | 87.5 (377/431) [84.0, 90.3] | 93.8 (823/877) [92.0, 95.3] | 52.9 (377/713) [49.1, 56.6] |
|  | Reader 3 | 64.8 (751/1159) [62.0, 67.5] | 90.7 (391/431) [87.6, 93.1] | 94.9 (751/791) [93.2, 96.3] | 48.9 (391/799) [45.4, 52.5] |
|  | Reader 4 | 87.6 (1015/1159) [85.5, 89.4] | 72.4 (312/431) [68.0, 76.4] | 89.5 (1015/1134) [87.6, 91.2] | 68.4 (312/456) [63.9, 72.6] |

Note—: Values are percentages (numerators/denominators) with [95% confidence intervals]. A *P*-value <0.05 indicates the statistically significant difference between LI-RADS v2018 and KLCA-NCC v2022. HCC = hepatocellular carcinoma; LI-RADS = Liver Imaging Reporting and Data System; KLCA-NCC = Korean Liver Cancer Association-National Cancer Center; PPV = positive predictive value; NPV = negative predictive value.

**Table S4. Performance of “definite HCC” in LI-RADS v2018 and KLCA-NCC v2022 in each reader**

| **Guidelines** | **Readers** | **Sensitivity (%)** | **Specificity (%)** | **PPV (%)** | **NPV (%)** |
| --- | --- | --- | --- | --- | --- |
| **LI-RADS v 2018** | Reader 1 | 70.5 (1195/1694) [68.3, 72.7] | 92.3 (693/751) [90.1, 94.0] | 95.4 (1195/1253) [94.0, 96.4] | 58.1 (693/1192) [55.2, 61.0] |
|  | Reader 2 | 69.8 (1182/1694) [67.5, 71.9] | 91.1 (684/751) [88.8, 92.9] | 94.6 (1182/1249) [93.2, 95.8] | 57.2 (684/1196) [54.3, 60.1] |
|  | Reader 3 | 58.1 (984/1694) [55.7, 60.4] | 92.5 (695/751) [90.4, 94.2] | 94.6 (984/1040) [93.1, 95.8] | 49.5 (695/1405) [46.8, 52.2] |
|  | Reader 4 | 83.8 (1419/1694) [81.9, 85.5] | 82.7 (621/751) [79.8, 85.2] | 91.6 (1419/1549) [90.1, 92.9] | 69.3 (621/896) [66.1, 72.3] |
| **KLCA-NCC v2022** | Reader 1 | 82.2 (1392/1694) [80.2, 84.0] | 87.4 (656/751) [84.7, 89.6] | 93.6 (1392/1487) [92.2, 94.8] | 68.5 (656/958) [65.3, 71.5] |
|  | Reader 2 | 77.7 (1316/1694) [75.6, 79.6] | 83.4 (626/751) [80.5, 85.9] | 91.3 (1316/1441) [89.8, 92.7] | 62.4 (626/1004) [59.2, 65.4] |
|  | Reader 3 | 64.6 (1095/1694) [62.3, 66.9] | 87.4 (656/751) [84.7, 89.6] | 92.0 (1095/1190) [90.3, 93.4] | 52.3 (656/1225) [49.4, 55.1] |
|  | Reader 4 | 88.2 (1494/1694) [86.5, 89.7] | 78.2 (587/751) [75.1, 81.0] | 90.1 (1494/1658) [88.6, 91.5] | 74.6 (587/787) [71.3, 77.6] |

Note—:Values are percentages (numerators/denominators) with [95% confidence intervals]. HCC = hepatocellular carcinoma; LI-RADS = Liver Imaging Reporting and Data System; KLCA-NCC = Korean Liver Cancer Association-National Cancer Center; PPV = positive predictive value; NPV = negative predictive value.

**Table S5. Comparison of the performance of LR-4 in LI-RADS and “probable HCC” in KLCA-NCC for diagnosing HCC in each reader**

| **Readers** | **PPV (%)** | | | |
| --- | --- | --- | --- | --- |
|  | LR-4 | “Probable HCC” | Difference | *P*-value |
| MRI (n = 2445) |  |  |  |  |
| Reader 1 | 73.9 (272/368) [69.1, 78.2] | 70.8 (119/168) [63.5, 77.2] | 3.1 [-3.2, 9.4] | 0.336 |
| Reader 2 | 68.5 (246/359) [63.5, 73.1] | 62.3 (66/106) [52.7, 70.9] | 6.3 [-2.7, 15.3] | 0.173 |
| Reader 3 | 66.7 (212/318) [61.2, 71.7] | 70.3 (104/148) [62.4, 77.1] | -3.6 [-11, 4.0] | 0.355 |
| Reader 4 | 61.2 (153/250) [54.8, 67.2] | 52.5 (84/160) [44.5, 60.3] | 8.7 [1.9, 15.5] | 0.012 |
| CT (n = 1590) |  |  |  |  |
| Reader 1 | 73.1 (68/93) [63.0, 81.3] | 88.2 (15/17) [63.2, 97.0] | -15 [-33, 2.5] | 0.093 |
| Reader 2 | 79.8 (67/84) [69.8, 87.0] | 100 (8/8) [-] | – | – |
| Reader 3 | 76.4 (113/148) [68.8, 82.5] | 100 (34/34) [-] | – | – |
| Reader 4 | 66.7 (72/108) [57.3, 74.9] | 65.0 (26/40) [49.2, 78.1] | 1.7 [-13, 15.9] | 0.819 |

Note—:Values are percentages (numerators/denominators) with [95% confidence intervals]. A *P*-value < 0.05 indicates statistically significance between LI-RADS v2018 and KLCA-NCC v2022. HCC = hepatocellular carcinoma; LI-RADS = Liver Imaging Reporting and Data System; KLCA-NCC = Korean Liver Cancer Association-National Cancer Center; PPV = positive predictive value; NPV = negative predictive value.

**Table S6. Proportion of HCC in LR-3 in LI-RADS v2018 and “indeterminate nodule” in KLCA-NCC v2022 in each reader**

| **Readers** | **Proportion of HCC (%)** | | | |
| --- | --- | --- | --- | --- |
|  | LR-3 | “indeterminate nodule” | Difference | *P*-value |
| MRI (n = 2445) |  |  |  |  |
| Reader 1 | 18.8 (9/48) [10.0, 32.4] | 40.7 (57/140) [32.6, 49.4] | -22.0 [-34.2, -9.8] | < 0.001 |
| Reader 2 | 14.7 (10/68) [8.0, 25.4] | 43.6 (78/179) [36.3, 51.2] | -28.9 [-39.6, -18.2] | < 0.001 |
| Reader 3 | 13.5 (15/111) [8.3, 21.3] | 44.8 (81/181) [37.5, 52.2] | -31.2 [-40.4, -22.1] | < 0.001 |
| Reader 4 | 7.2 (5/69) [3.0, 16.3] | 28.6 (28/98) [20.3, 38.6] | -21.3 [-31.2, -11.5] | < 0.001 |
| Pooled data | 13.2 (39/296) [9.0, 18.9] | 40.8 (244/598) [34.6, 47.4] | -27.6 [-34.5, -20.8] | < 0.001 |
| CT (n = 1590) |  |  |  |  |
| Reader 1 | 52.5 (85/162) [44.4, 60.4] | 41.1 (177/431) [36.3, 46.0] | 11.4 [5.3, 17.5] | < 0.001 |
| Reader 2 | 61.9 (166/268) [55.9, 67.6] | 50.8 (241/474) [46.2, 55.4] | 11.1 [7.1, 15.1] | < 0.001 |
| Reader 3 | 57.7 (127/220) [50.9, 64.2] | 53.6 (262/489) [49.0, 58.1] | 4.1 [-0.8, 9.1] | 0.103 |
| Reader 4 | 23.6 (33/140) [16.9, 31.9] | 33.8 (97/287) [28.4, 39.7] | -10.2 [-16.0, -4.5] | < 0.001 |
| Pooled data | 52.0 (411/790) [46.2, 57.8] | 46.2 (777/1681) [41.9, 50.6] | 5.8 [2.3, 9.3] | 0.001 |

Note—:Values are percentages (numerators/denominators) with [95% confidence intervals]. A *P*-value < 0.05 indicates statistically significance between LI-RADS v2018 and KLCA-NCC v2022. HCC = hepatocellular carcinoma; LI-RADS = Liver Imaging Reporting and Data System; KLCA-NCC = Korean Liver Cancer Association-National Cancer Center; PPV = positive predictive value; NPV = negative predictive value.

**Table S7. Impactive of extended washout timing in LR-4 observations for diagnosing HCC on MRI**

| **Guidelines** | **Sensitivity (%)** | **Specificity (%)** | **PPV (%)** | **NPV (%)** |
| --- | --- | --- | --- | --- |
| MRI (n = 2445) | | | | |
| LR-5 | 70.5 (4780/6776) [68.8, 72.3] | 89.6 (2693/3004) [87.8, 91.2] | 93.9 (4780/5091) [92.7, 94.9] | 57.4 (2693/4689) [54.7, 60.1] |
| LR-5, LR-4 with both non-rim APHE and TP or HBP hypointensity | 77.7 (5268/6776) [76.1, 79.3] | 84.9 (2550/3004) [82.7, 86.8] | 92.1 (5268/5772) [90.8, 93.2] | 62.8 (2550/4058) [60.1, 65.5] |
| Difference | 7.2 [6.2, 8.2] | -4.8 [-5.9, -3.6] | -1.8 [-2.4, -1.3] | 5.4 [4.4, 6.4] |
| *P*-value | <0.001 | <0.001 | <0.001 | <0.001 |

Note—: Data are pooled results across four readers (e.g., 2445 × 4 = 9780 lesions). Values are percentages (numerators/denominators) with [95% confidence intervals]. A *P*-value <0.05 indicates statistically significant difference. HCC= hepatocellular carcinoma; PPV = positive predictive value; NPV = negative predictive value; APHE = arterial phase hyperenhancement; TP = transitional phase; HBP = hepatobiliary phase

**Table S8. Interobserver agreement on CT and MRI**

| **Diagnostic category** | **MRI** | | **CT** | |
| --- | --- | --- | --- | --- |
|  | Fleiss kappa | Gwet’s AC1 | Fleiss kappa | Gwet’s AC1 |
| **LI-RADS v2018** | | | | |
| LR-5 | 0.637 [0.617, 0.658] | 0.799 [0.736, 0.862] | 0.654 [0.628, 0.680] | 0.809 [0.751, 0.867] |
| LR-4 | 0.533 [0.499, 0.566] | 0.887 [0.874, 0.901] | 0.252 [0.207, 0.297] | 0.902 [0.877, 0.927] |
| LR-3 | 0.354 [0.289, 0.420] | 0.962 [0.947, 0.977] | 0.442 [0.401, 0.483] | 0.872 [0.821, 0.922] |
| **KLCA-NCC v2022** | | | | |
| “Definite HCC” | 0.653 [0.632, 0.674] | 0.778 [0.670, 0.886] | 0.643 [0.617, 0.669] | 0.770 [0.699, 0.841] |
| “Probable HCC” | 0.425 [0.375, 0.476] | 0.932 [0.913, 0.951] | 0.032 [0.001, 0.063] | 0.970 [0.950, 0.989] |
| “Indeterminate nodule” | 0.608 [0.586, 0.631] | 0.769 [0.656, 0.882] | 0.650 [0.624, 0.676] | 0.777 [0.704, 0.850] |

Note—: Data in bracket are 95% CI.

**REFERENCE**

1.	Yoon JH, Kim YK, Kim JW, et al (2024) Comparison of Four Diagnostic Guidelines for Hepatocellular Carcinoma Using Gadoxetic Acid-enhanced Liver MRI. Radiology 311:e233114. https://doi.org/10.1148/radiol.233114

2.	Yoon JH, Chang W, Kim YK, et al (2025) Comparison of Gadoxetic Acid-Enhanced Liver Magnetic Resonance Imaging and Contrast-Enhanced Computed Tomography for the Noninvasive Diagnosis of Hepatocellular Carcinoma. Liver Cancer 1–13. https://doi.org/10.1159/000545965

3.	Chernyak V, Fowler KJ, Do RKG, et al (2023) LI-RADS: Looking Back, Looking Forward. Radiology 307:e222801. https://doi.org/10.1148/radiol.222801

4.	Korean Liver Cancer Association (KLCA) and NCC (NCC) Korea (2022) 2022 KLCA-NCC Korea Practice Guidelines for the Management of Hepatocellular Carcinoma. Korean J Radiol 23:1126–1240. https://doi.org/10.3348/kjr.2022.0822

5.	Reizine E, Mulé S, Luciani A (2022) Focal Benign Liver Lesions and Their Diagnostic Pitfalls. Radiol Clin North Am 60:755–773. https://doi.org/10.1016/j.rcl.2022.05.005

6.	Kim JH, Yoon JH, Joo I, Lee JM (2021) Evaluation of Primary Liver Cancers Using Hepatocyte-Specific Contrast-Enhanced MRI: Pitfalls and Potential Tips. JMRI 53:655–675. https://doi.org/10.1002/jmri.27213

7.	Yoneda N, Matsui O, Kitao A, et al (2016) Benign Hepatocellular Nodules: Hepatobiliary Phase of Gadoxetic	Acid–enhanced MR Imaging Based on Molecular Background. RadioGraphics 36:2010–2027. https://doi.org/10.1148/rg.2016160037

8.	Nolan P, Catania R, Vendrami C, et al (2022) Large Regenerative Nodules and Focal Nodular Hyperplasia-Like Lesions. Radiol Clin North Am 60:795–808. https://doi.org/10.1016/j.rcl.2022.05.004

9.	Kim Y-K, Kim CS, Moon WS, et al (2005) MRI Findings of Focal Eosinophilic Liver Diseases. AJR 184:1541–1548. https://doi.org/10.2214/ajr.184.5.01841541

10.	You S-H, Park BJ, Kim YH (2017) Hepatic Lesions that Mimic Metastasis on Radiological Imaging during Chemotherapy for Gastrointestinal Malignancy: Recent Updates. KJR 18:413–426. https://doi.org/10.3348/kjr.2017.18.3.413
